# Effects of Rigid and Nonrigid Connections between the Miniscrew and Anchorage Tooth on Dynamics, Efficacy, and Adverse Effects of Maxillary Second Molar Protraction: A Finite Element Analysis

**DOI:** 10.1155/2022/4714347

**Published:** 2022-10-14

**Authors:** Marzieh Mazhari, Mehrnaz Moradinejad, Mohsen Mazhary, Atefe Rekabi, Vahid Rakhshan

**Affiliations:** ^1^Department of Orthodontics, School of Dentistry, Ahvaz Jundishapur University of Medical Sciences, Ahvaz, Iran; ^2^Department of Civil and Environmental Engineering, ACECR Institute for Higher Education, Ahvaz, Iran; ^3^Department of Anatomy, Dental School, Azad University of Medical Sciences, Tehran, Iran

## Abstract

**Introduction:**

Direct, rigid indirect, and nonrigid indirect absolute anchorages using temporary anchorage devices (TADs, mini-implants/miniscrews) can provide promising opportunities for challenging, yet common, orthodontic tooth movements such as molar protraction. Rigid rectangular wire and ligature wire are the most common methods of attaching a tooth to a miniscrew in indirect anchorages. We aimed to provide a comparison of the rigidity of the connecting wire in terms of stress on the miniscrew, the anchorage loss, and the risk of root resorption using finite element analysis (FEA).

**Methods:**

The maxillary right second molar was protracted into the proximal space at a 150 g load (1) using direct absolute anchorage with a tapered miniscrew implanted between the premolar roots and using indirect absolute anchorage with the second premolar reinforced by the miniscrew through (2) a rigid stainless steel (SS) wire or (3) a nonrigid SS ligature wire (4) at different elastic moduli. Stresses and displacements of 4 models' elements were measured. The risk of external root resorption was evaluated.

**Results:**

Connecting the tooth to the miniscrew using rigid full-size wire (model 2) compared to ligature (model 3) can give better control of the anchorage (using the ligature wire, the anchorage loss is 1.5 times larger than the rectangular wire) and may reduce the risk of root resorption of the anchorage unit. However, the risk of miniscrew failure increases with a rigid connection, although it is still lower than with direct anchorage. The miniscrew stress when using a ligature is approximately 30% of the rigid model using the rectangular wire. The miniscrew stress using the rectangular wire is approximately 82.4% of the miniscrew stress in the direct model. Parametric analysis shows that the higher the elastic modulus of the miniscrew-tooth connecting wire in the indirect anchorage, the less the anchorage loss/palatal rotation of the premolars/and the risk of root resorption of the anchorage teeth and instead the stress on the miniscrew increases.

**Conclusions:**

Direct anchorage (followed by rigid indirect anchorage but not nonrigid) might be recommended when the premolars should not be moved or premolar root resorption is a concern. Miniscrew loosening risk might be the highest in direct anchorage and lowest in nonrigid indirect anchorage (which might be recommended for poor bone densities).

## 1. Introduction

In orthodontics, the first permanent molar is the key to occlusion [1]. This tooth appears in the mouth at the age of 6 and is claimed to be the most commonly missing tooth in adults [2]. Losing this tooth will cause numerous problems such as the disruption of arch symmetry, drifting of the neighboring teeth into its space, malocclusion, and temporomandibular joint problems [3]. There are several solutions to restore the function of this missing tooth, including fixed partial dentures, dental implants, or orthodontic replacement with a second molar if sufficient anchorage is available [4]. Molar protraction and proper anchorage may be needed also in other clinical conditions such as the unforeseen residual space after aligning the teeth, the congenital missing of the second premolars, and the extraction of hopeless teeth [5]. Therefore, various techniques have been proposed for obtaining proper anchorages for molar protraction.

Anchorage is a critical part and a prerequisite of orthodontic treatments [6–8]; and anchorage loss is a serious complication [9, 10]. When the anchorage unit consists of only teeth, it faces limitations and conditions similar to the movement unit and may move like an active unit under the influence of force; therefore, the orthodontist should strengthen the anchorage unit [11–13]. Different methods and appliances have been proposed for strengthening the anchorage unit, such as extraoral anchorages or cortical anchorages [5, 14, 15]. The use of nondental structures as anchorage units allows therapeutic movements or growth modifications to be performed without side effects [5]. It has been proven that implants can be a reliable and effective tool as orthodontic anchorage and have created a new pattern of anchorage called absolute anchorage [5, 16, 17]. A common form of absolute anchorages is utilizing mini-implants/miniscrews [18].

Miniscrews are gaining ever-increasing popularity among orthodontists, as their use as an anchorage unit dramatically improves the balance between the active unit and the anchorage unit, and can have significant therapeutic benefits [18]. Miniscrews can provide two types of absolute anchorage: direct anchorage and indirect anchorage. In the direct anchorage method, the force from the miniscrew is directly applied to the teeth of the active unit [19–22]. In the indirect absolute anchorage, the anchorage unit is consisted of teeth and the orthodontic force is applied from the teeth in the anchorage unit to the active unit; however, the teeth in the anchorage unit are reinforced and immobilized using a miniscrew [22–25]. In this method, the miniscrew can be placed either in the interradicular space or somewhere else such as the palate or retromolar area in the mandible, depending on the available space, the used materials, and the dynamics of orthodontic force [23]. Indirect anchorage allows the miniscrew to be placed in a variety of positions and reduces the risk of root trauma; other advantages of this type of anchorage include the use of standard orthodontic methods in the application of force, which provides reliable control over tooth movement [19, 22, 26, 27].

In the indirect absolute anchorage method, anchorage unit teeth can be fixed with rigid components such as stainless steel (SS) wires or nonrigid components such as SS ligatures [23, 28]. In the use of rigid components such as SS wires, the miniscrew can be placed in any location regardless of the direction of force, because this structure can act as a tie and strut, and there is more freedom in choosing the location of the miniscrew, and hence, the operator can focus more on choosing the ideal anatomical location of the implant [23].

Failure rate of miniscrews may be rather high (about 15% to 20%) in the direct absolute anchorage method [29], but may be lower in the indirect method with lower miniscrew loads [26]. Direct anchorage can have more side effects than indirect anchorage in challenging clinical situations such as molar protraction (for example, mesial rotation during molar protraction) due to the torsional moment caused by laterally exerted force [30]. Despite numerous benefits of indirect anchorage, the risk of anchorage loss in this method is unknown [30, 31].

In spite of the increasing use of miniscrews, there is still insufficient knowledge about the optimal placement patterns, safety, and mini-implant anchorage characteristics in relation to the surrounding bone. This lack of information can lead to a high failure rate of this device and be a major deterrent to their use [31]. There are different methods for molar protraction using interradicular miniscrews, and the use of each of these methods may lead to different results (in terms of miniscrew stability and the amount and type of tooth movement). However, the role of the type of wire connecting the miniscrew to the anchorage unit teeth in indirect anchorage has not been investigated until now.

Rigid rectangular wire or ligature wire are the most common methods of attaching a tooth to a miniscrew in indirect anchorage. Moreover, as stated above, it seems that no study has assessed the effects of rigidity of the tooth-implant connecting wire in terms of the stress exerted on the miniscrew, the amount of anchorage loss, and the risk of root resorption (which can be caused by orthodontic tooth movement [32]). Therefore, we compared the connection of the anchorage tooth with the miniscrew using the full-size rectangular rigid, with a third model (direct anchorage method) serving as the gold standard or control model. We measured the effects of the type of connector wire on the stability of the miniscrew and the movement of the anchorage unit. In this way, it is possible to choose the most appropriate technique for each clinical situation, depending on the characteristics of occlusion and bone quality.

## 2. Materials and Methods

This was a 4-phase experimental in silico simulation study. The model designed in this study was a simulation of the clinical protraction of the right maxillary second molar into the extraction space of the right first molar. A titanium square-threaded tapered miniscrew with dimensions of 8 × 1.6 mm with a head length of 2 mm [4] was placed in the buccal and distal sides of the first premolar perpendicular to the bone surface.

A stainless steel (SS) archwire 0.019′ × 0.025′ was simulated as the base archwire in all models. The connection between the miniscrew and the bone was defined as a tight tie in all models. A protractive force of 150 g was applied within each model. The spring type used in this study was SS closed-coil [33] with wire diameter, lumen size, initial length range, estimated stiffness of 0.010 (inch), 0.030 (inch), 4-10 (mm), and 1 (N/Sq·mm), respectively.

### 2.1. Study Models

**Figure 1:**
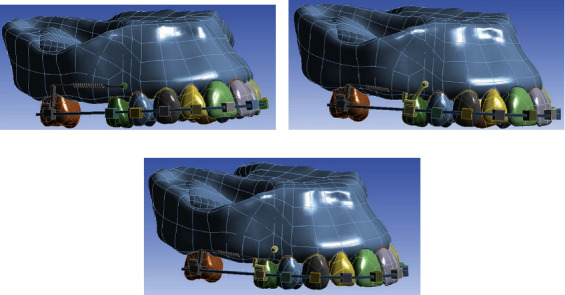
The three different models used in this study: (a) model 1 with direct anchorage; (b) model 2 with rigid indirect anchorage; (c) model 3 with nonrigid indirect anchorage.

Based on the above model, four models (three main models and one extension) were prepared with some differences (Figure 1).

#### 2.1.1. Model 1 (Direct Absolute Anchorage)

Model 1 is the model with direct connection between the second molar and the miniscrew using a spring between the power arm of the molar tooth and the mini screw.

#### 2.1.2. Model 2 (Rigid Indirect Absolute Anchorage)

In this model, a spring was used to connect the second molar to the second premolar; using a rectangular 0.021′ × 0.025′ stainless steel (SS) wire, the second premolar was engaged with the miniscrew (rigid connection).

#### 2.1.3. Model 3 (Nonrigid Indirect Absolute Anchorage)

In this model, again a spring was used to connect the second molar to the second premolar; however, the second premolar was engaged with the miniscrew using a 0.5 mm SS ligature wire as the nonrigid connection (which had a smaller elastic modulus and yield stress compared to the SS rectangular wire used in model 2). In the direct anchorage model (model 1), a force of 150 g [4] was applied parallel with the occlusal plane using a spring between the center of the miniscrew head and the 8 mm long SS power arm of the molar band. In the two indirect anchorage models (models 2 and 3), the second premolar and miniscrew were attached using a 0.021′ × 0.025′ SS wire (in the rigid indirect model [model 2]) and a 0.5 mm SS ligature (in the nonrigid indirect model [model 3]). In both the indirect models, a protraction force of 150 g was applied to the second molar using a spring between the molar's hook and the second premolar's hook (Figure 1).

#### 2.1.4. Model 4 (Parametric Extensions of Model 3)

Since various elastic moduli had been stated for the ligature wire in the literature [34–40], we also simulated a range of elastic moduli in model 3 and reported the effects of parametric changes in the ligature wire rigidity on stresses and displacements of model elements.

**Figure 2:**
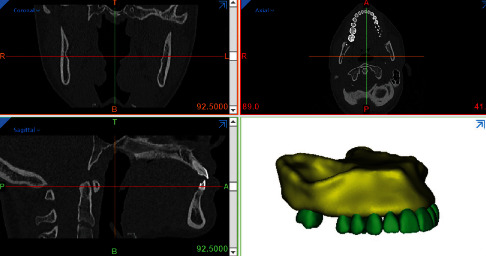
Creating the 3D model of the maxilla.

**Figure 3:**
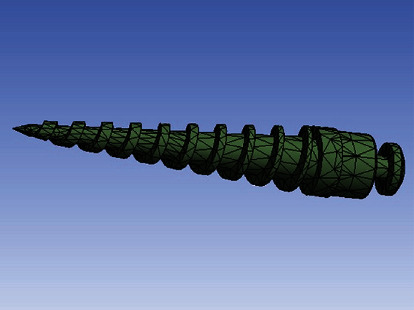
The miniscrew in use.

**Figure 4:**
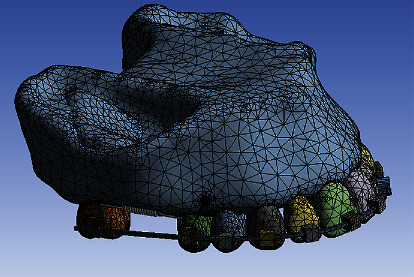
An example of meshing.

The bone, tooth, and PDL models were modeled in Mimics 3D image processing software (Mimics Research 21; Materialise NV; Brussels, Belgium) and 3-Matic (Materialise). First, 16-bit monochrome CT scan images of a young man with a distance of 1 mm between the slices and a resolution of 768 × 768 (NewTom VGi; Finland) were entered into Mimics. Using segmentation tools, masks for the maxilla, PDLs, teeth, and bones were created, and then a 3D model of these components was created using the Calculate 3D command (Figure 2). Then, all the parts were exported in the “.stl” format from these softwares. The miniscrews were designed with the help of Helix and Revolution commands in Solidworks software (version 2018, Dassault Systemes; Paris, France), and the brackets and orthodontic wires were designed in ANSYS software (ANSYS Workbench 2021, ANSYS Inc., Canonsburg, Pennsylvania, USA). The components were assembled together in ANSYS (Figure 3). Parts exported in the “.stl” format from Mimics and 3-Matic softwares (Materialise) in Geomagic software (3D Systems, Morrisville, North Carolina, United States) became “parts” in the “.stp” format. After converting all geometries to the “.stp” format, these geometries were entered into Ansys Workbench 2021 (ANSYS Inc) for analysis. The maxilla was fixed at its upper surface. There were 487540 nodes, 227394 contact elements, 254167 solid elements, and 481564 total elements (Figure 4). The finite element approximation was of higher order (quadratic functions were used). Materials in the 3 models were assigned the properties in Table 1 [41–45].

### 2.2. Outcomes

The created and loaded models were compared in terms of von Mises stresses, hydrostatic stresses, and movements of all the involved elements. If the PDL hydrostatic pressure surpasses the capillary pressure in the area, the risk of root resorption will increase owing to the impaired blood flow. PDL capillary pressure might be about 0.002 to 0.005 MPa [46]. Also, compressive hydrostatic stresses at the PDLs were compared with 0.0047 MPa as a threshold for significant increase of the risk of external root resorption [46, 47].

## 3. Results

### 3.1. Model Stresses

**Figure 5:**
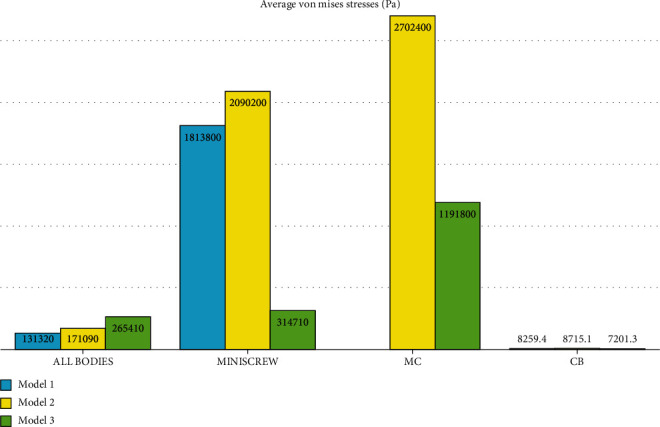
Average stresses exerted to different components in different models. MC: miniscrew+connections; CB: cancellous bone. Model 1 has direct anchorage; model 2 has rigid indirect anchorage; model 3 has nonrigid indirect anchorage.

**Figure 6:**
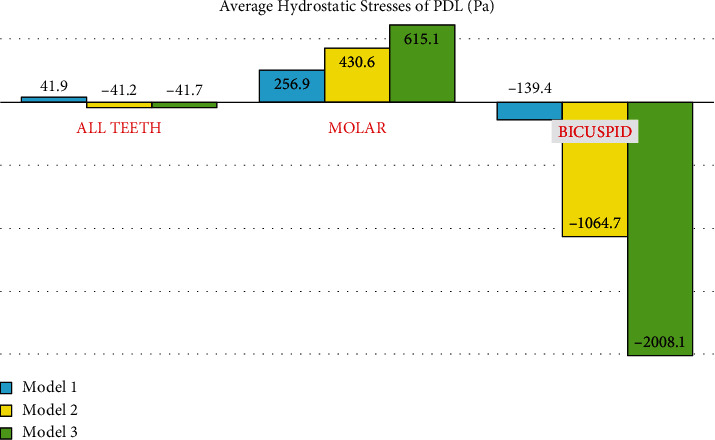
Average hydrostatic pressures exerted to the PDLs in different models. Positive values are tensile stresses, and negative values are compressive pressures. Model 1 has direct anchorage; model 2 has rigid indirect anchorage; model 3 has nonrigid indirect anchorage.

**Figure 7:**
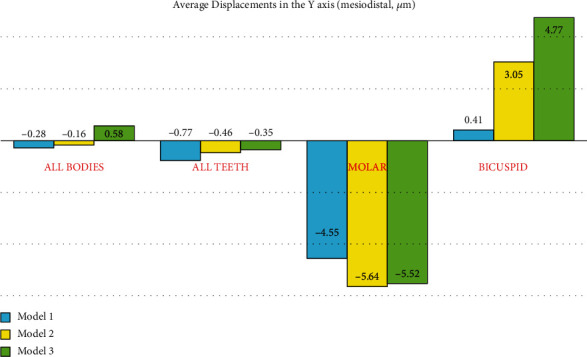
Average displacements in the *Y*-axis (the mesiodistal direction, *μ*m) in different models. Model 1 has direct anchorage; model 2 has rigid indirect anchorage; model 3 has nonrigid indirect anchorage. Positive values indicate distalization while negative values indicate mesialization.

**Figure 8:**
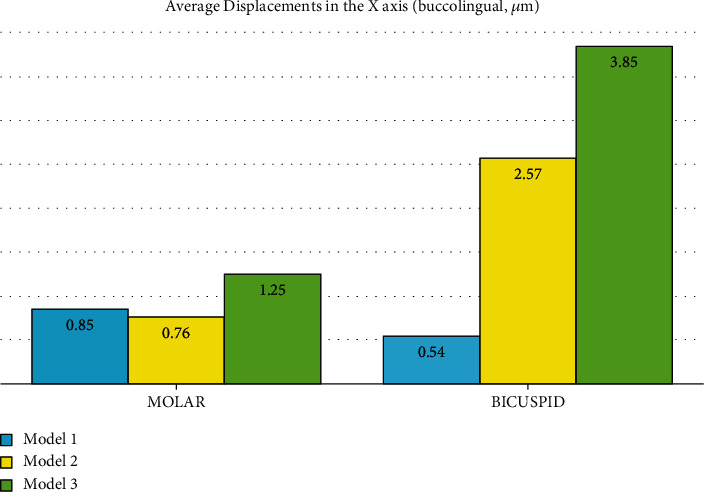
Average displacements in the *X*-axis (the buccolingual direction, *μ*m) in different models. Model 1 has direct anchorage; model 2 has rigid indirect anchorage; model 3 has nonrigid indirect anchorage. Negative values indicate buccal movement, while positive values indicate palatalization.

**Figure 9:**
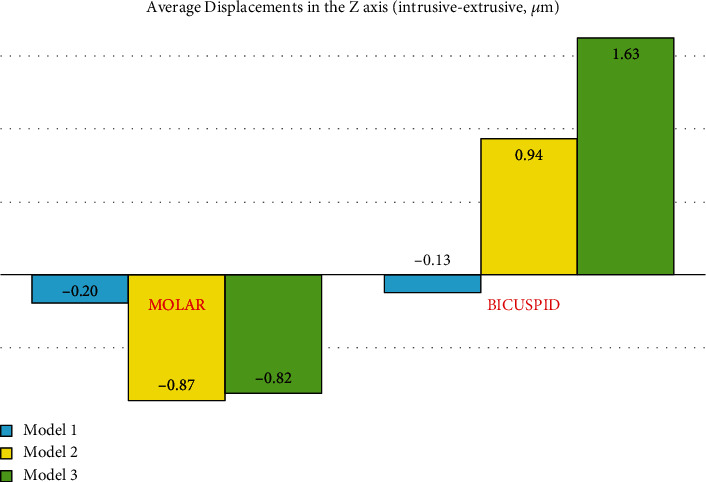
Average displacements in the *Z*-axis (the intrusive-extrusive direction, *μ*m) in different models. Model 1 has direct anchorage; model 2 has rigid indirect anchorage; model 3 has nonrigid indirect anchorage. Positive values mean intrusive movement, while negative values mean extrusion.

**Figure 10:**
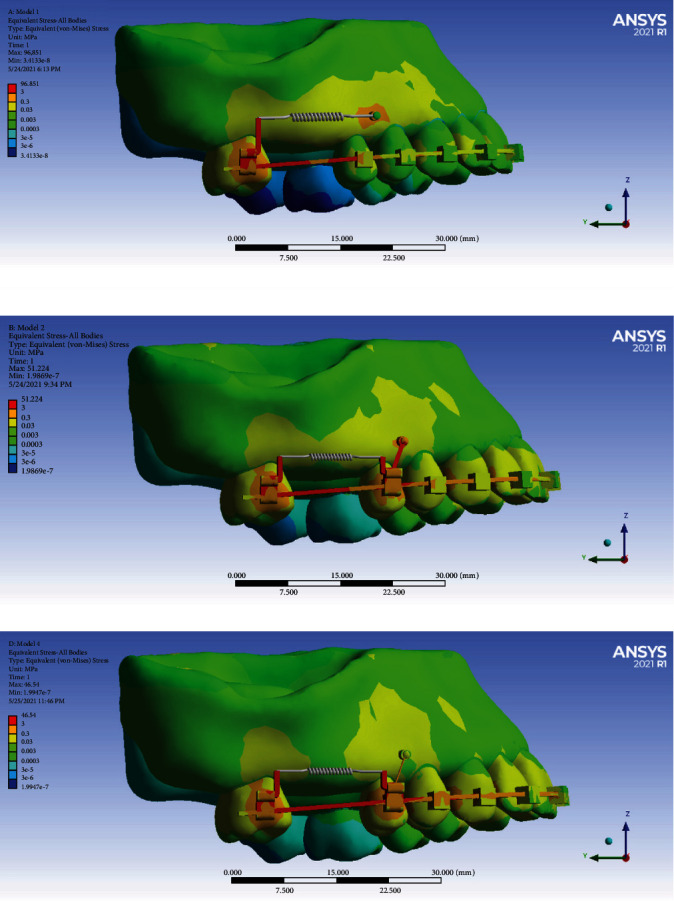
Stresses of the model parts in different models: (a) model 1 with direct anchorage; (b) model 2 with rigid indirect anchorage; (c) model 3 with nonrigid indirect anchorage.

Overall, the stress was distributed mostly in the molar band's power arm followed by the buccal surface of the molar, around the miniscrew, and the buccal surface of premolars and the buccal palate of the alveolar bone of the molars, premolars, and canine (Figures 5–10). The pattern of stress differed between Model 1 (direct anchorage) with Models 2 and 3 (indirect anchorages), in a way that the stress of premolars was considerably greater in the indirect anchorage models (Figures 5–10). The extents of the maximum stress were much greater in the direct anchorage model than the two indirect anchorage models (Table 2). The average stress of the whole system was greater in Model 3 followed by Models 2 and 1 (Table 2).

MC: miniscrew+connections; CB: cancellous bone.

### 3.2. Miniscrew Stresses

**Figure 11:**
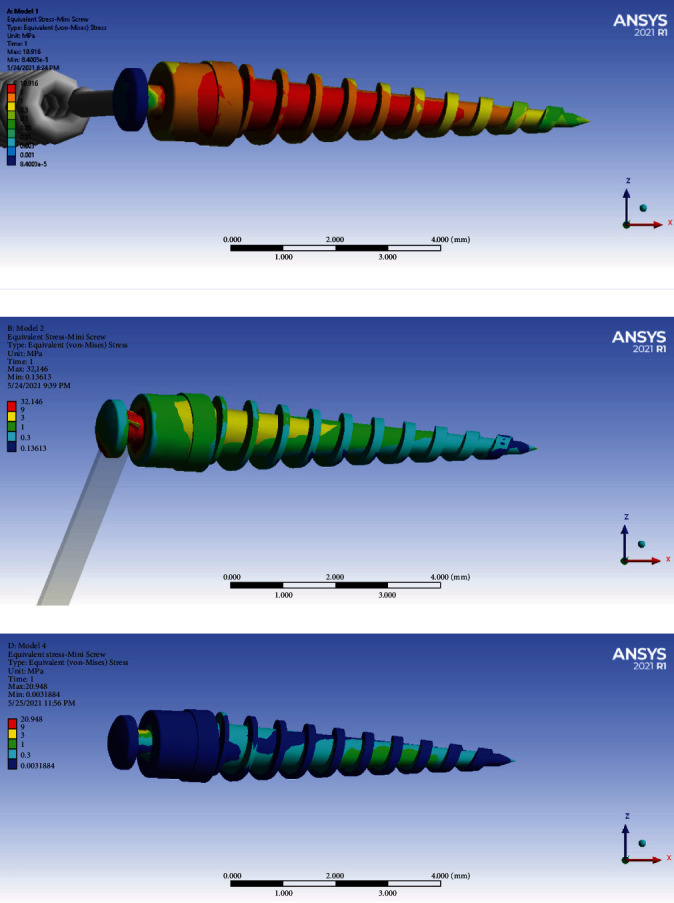
Stresses of the miniscrew in different models: (a) model 1 with direct anchorage; (b) model 2 with rigid indirect anchorage; (c) model 3 with nonrigid indirect anchorage.

The miniscrew stress when using a ligature is approximately 30% of the rigid model using the rectangular wire. The miniscrew stress using the rectangular wire is approximately 82.4% of the miniscrew stress in the direct model. As seen in Figure 11(a), the highest amount of stress in the body of the miniscrew was created in the direct anchorage model and its magnitude was 10.916 MPa.  Figure 11(b) shows the miniscrew in the rigid indirect anchorage model. The maximum stress in the body of the miniscrew in this model was 9 MPa and in the cervical half of this miniscrew.  Figure 11(c) shows the miniscrew in the nonrigid indirect anchorage model. The maximum stress in the body of the miniscrew in this model was 3 MPa (green color spectrum). According to the above results, the highest stress was applied to the screw in the direct anchorage model, and the lowest stress was applied in the indirect nonrigid anchorage model (Table 2).

### 3.3. Cancellous Bone Stresses

**Figure 12:**
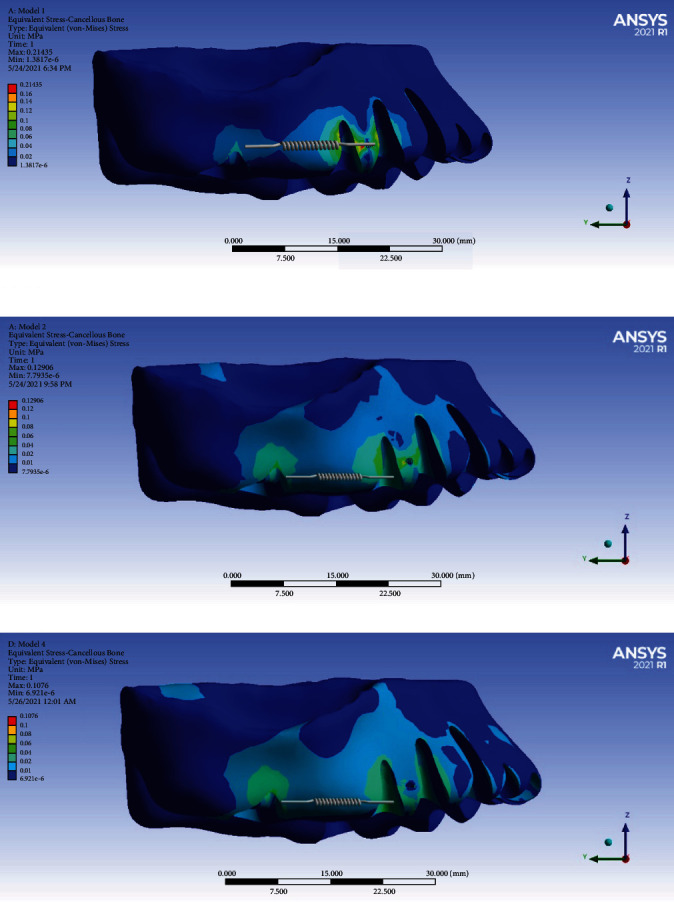
Stresses of the bone in different models: (a) model 1 with direct anchorage; (b) model 2 with rigid indirect anchorage; (c) model 3 with nonrigid indirect anchorage.

The stress on the spongy bone was almost halved in the rigid model (0.12906 MPa) and the nonrigid model (0.1076 MPa) compared to the direct model (0.21435 MPa). In Figure 12(a), the stress exerted to the spongy bone is shown in the direct anchorage model. At the location of the miniscrew socket, the color is red, which indicates the maximum stress created on the spongy bone (with a magnitude of 0.21435 MPa) due to the application of force.  Figure 12(b) shows the stress on the spongy bone mostly around the second premolar and second molar roots in the rigid indirect anchorage model. The maximum stress reported in this model was 0.12906 MPa.  Figure 12(c) shows the equivalent stress on the spongy bone in the nonrigid indirect anchorage model. The maximum stress reported in this model was 0.1076 MPa which would be observed around the second premolar and second molar roots. As can be seen from the Table 2, the highest amount of stress in the spongy bone was created in the direct anchorage model (Figure 12(a)) and was in the miniscrew hole followed by the bone surrounding the second premolar root, while the lowest amount of stress was seen in the spongy bone in the indirect nonrigid anchorage model 3 (Figure 12(c), Table 2).

### 3.4. Hydrostatic Pressure at the Premolar PDL

**Figure 13:**
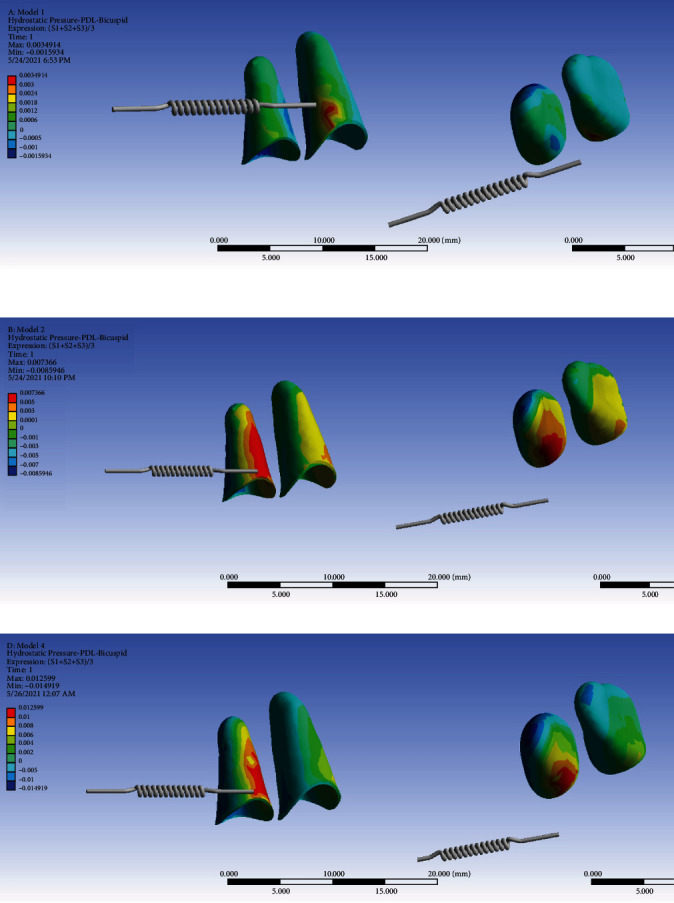
Hydrostatic stresses in the PDL of the premolars in different models: (a) model 1 with direct anchorage; (b) model 2 with rigid indirect anchorage; (c) model 3 with nonrigid indirect anchorage. Negative values indicate compressive stresses, and positive values indicate tensile ones. Negative values smaller than -0.0047 MPa pose a significantly higher external root resorption risk. The spring shows the force direction. The pair of root PDLs on the right side of each subimage is exactly the same pair of root PDLs on the left side, only from a different angle of view (apical view).

Using a ligature wire, more root resorption was observed in the anchorage unit.  Figure 13(a) shows the hydrostatic stress in the PDL of the premolars in the direct anchorage model. The red color spectrum indicates the highest amount of tensile hydrostatic stress created in the PDL of cervical third of the distobuccal side of the root of the first premolar at 0.0035 MPa. The maximum tensile strength is seen over a small area close to the miniscrew and may be caused by the tension caused by the traction of miniscrew to the distal side.  Figure 13(b) shows the hydrostatic stress in the PDL of premolars in the rigid indirect anchorage model. The red color spectrum in the PDL of the second premolar root indicates the maximum tensile stress created at 0.007366 MPa on the buccal side extending to the mesial side through the cervical to the apical areas. The maximum tensile hydrostatic stress was as well seen on the buccal side of the PDL of the first premolar root.  Figure 13(c) shows the hydrostatic stress in the PDL of premolars in the nonrigid indirect anchorage model. Similar to the pattern of the tensile strength observed in model 2, the maximum amount of tensile stress is created at 0.012599 MPa in the root of the second premolar over the buccal and mesial sides from the cervical to the apical areas. Unlike model 2, in this model, the tensile hydrostatic stress is much less in the first premolar PDL. In the comparison of these three models, the lowest amount of tensile hydrostatic stress in the premolars was related to the direct anchorage model, while the highest hydrostatic pressure was seen in the nonrigid indirect anchorage model (0.012599 MPa). In the two indirect anchorage models, the compressive hydrostatic pressure (shown by the color blue) is seen on the distal sides of the roots, and is considerably greater in the nonrigid indirect model (model 3). In the direct anchorage model (model 1), the compressive hydrostatic pressure (blue) was observed in the mesial surface of the second premolar root, right beside the miniscrew hole, suggesting that it is the miniscrew that is exerting the compressive force over the root of the second premolar (Figure 13, Table 2).

### 3.5. Hydrostatic Stress in the Second Molar PDL

In the rigid model, using the rectangular wire, the maximum compressive stress was observed in the molar. The reason for the slight difference with the nonrigid model seems to be the greater extent of distal movement of the premolars. In fact, with more movement of the premolars in the distal direction in the third model (nonrigid) compared to the second one (rigid), the spring closes and the force decreases.

**Figure 14:**
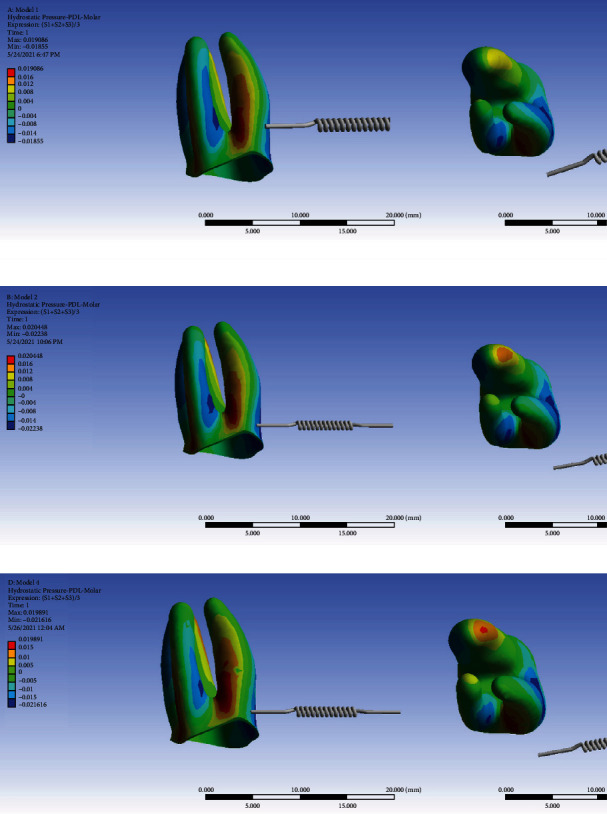
Hydrostatic stresses in the PDL of the second molar in different models: (a) model 1 with direct anchorage; (b) model 2 with rigid indirect anchorage; (c) model 3 with nonrigid indirect anchorage. Negative values indicate compressive stresses; positive values indicate tensile stresses. Negative values smaller than -0.0047 MPa pose a considerably higher risk for root resorption. The spring shows the force direction. The root PDLs on the right side of each subimage are exactly the same PDLs on the left side, only from a different angle of view (apical view).

The patterns of hydrostatic pressure distribution in the molar root PDLs were similar in the three models, with the tensile stress (warm colors) being higher in the distal sides of the mesiobuccal and distobuccal roots and the mesial side of the palatal root (which may be due to a mesial-in rotation). The amounts of the maximum tensile stress were rather similar among the models (0.019086, 0.020448, and 0.019891 MPa, respectively, in models 1, 2, and 3). On the other hand, the compressive hydrostatic pressure (blue color spectrum) was seen on the mesial sides of the buccal roots, on the mesial side of the root trunk, and on the distal side of the palatal root, reinforcing the “mesial-in rotation” idea. The compressive stress was the greatest in model 2 and the smallest in model 1 (Figure 14, Table 2).

### 3.6. Displacement through the *Y*-Axis (Mesiodistal)

#### 3.6.1. The Premolars

**Figure 15:**
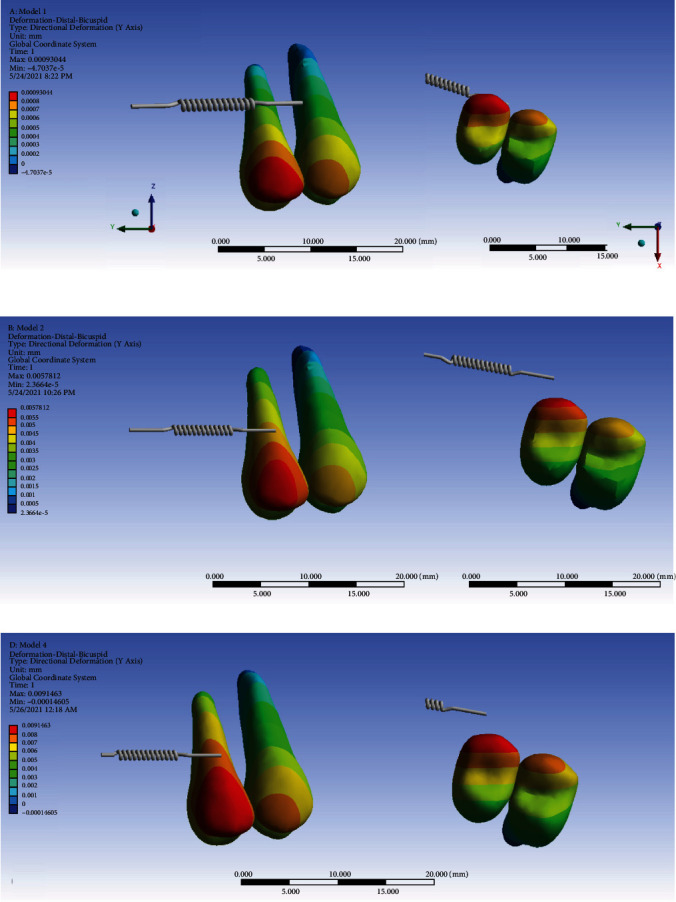
Displacements of the premolars through the *Y*-axis (the mesiodistal direction) in different models: (a) model 1 with direct anchorage; (b) model 2 with rigid indirect anchorage; (c) model 3 with nonrigid indirect anchorage. Positive values indicate distalization; negative values mean mesialization. The spring shows the force direction.

Using the ligature wire, the anchorage loss was 1.5 times the amount of anchorage loss using the rectangular wire. The patterns of displacement in the mesiodistal direction were similar among the models (especially between models 1 and 2). Positive values indicate distal movement while negative values indicate mesial movement. The most extent of distal displacement was seen in the buccal side of the crown of the first premolar followed by the crown of the second premolar. Apical areas displaced less than the coronal areas, indicating tipping of these teeth. Also, palatal sides moved less than buccal sides, indicating some degree of rotation as well. The maximum displacement extents were the highest in model 3 and the lowest in model 1. Comparing the extents of movement in the distal direction, the displacement of the premolars in the nonrigid indirect anchorage model was the highest extent (highest anchorage loss) and it had the lowest extent in the direct anchorage model (the lowest anchorage loss) (Figure 15, Table 2).

#### 3.6.2. The Second Molar

In the direct model, the force is applied close to the center of resistance of the molar, which leads to a uniform distribution of stress throughout the tooth. Therefore, the movement will be bodily, and compared to models 2 and 3 (in which the force is applied at a distance from the center of resistance and tipping is done), less movement is observed in the direct model. In the nonrigid model using the ligature wire, more distal movement of the premolars is observed compared to the second model (in which, the movement of the premolars is inhibited using the rectangular wire); the more distal movement of the premolars leads to spring closure and reduced force. Thus, using the ligature wire, the molar's displacement is slightly reduced (second model: 0.00564 mm, third model: 0.00552 mm).

**Figure 16:**
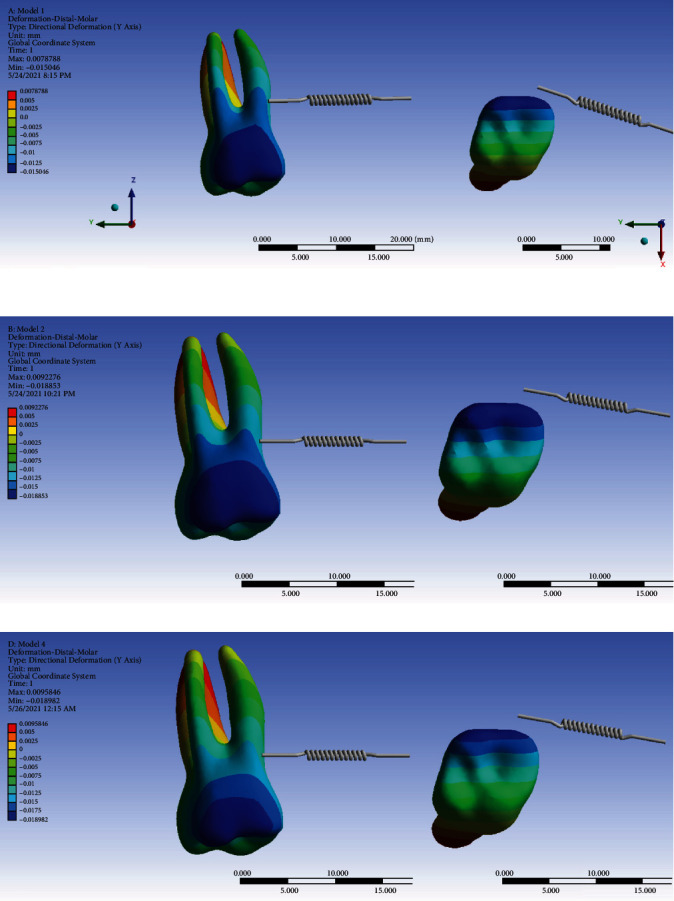
Displacements of the second molar on the *Y*-axis (the mesiodistal direction) in different models: (a) model 1 with direct anchorage; (b) model 2 with rigid indirect anchorage; (c) model 3 with nonrigid indirect anchorage. Negative values indicate mesialization. The spring shows the force direction.

The pattern of second molar mesialization was similar in all three models, with the buccal side being mesialized more than the palatal side, which indicates a “mesial-in” rotation of the tooth during protraction. Also, the coronal mesialization was greater than apical movement (which became slightly distalized), indicating an uncontrolled tipping movement. The amounts of maximum mesialization were similar for the indirect anchorage models (-0.018853 and -0.018982 mm, respectively, in models 2 and 3), both being greater than the extent of mesialization in the direct model (model 1, -0.015046 mm). The same pattern was also seen in the amounts of the average mesialization (Figure 16, Table 2).

### 3.7. Displacement on the *X*-Axis (Buccolingual)

#### 3.7.1. The Premolars

**Figure 17:**
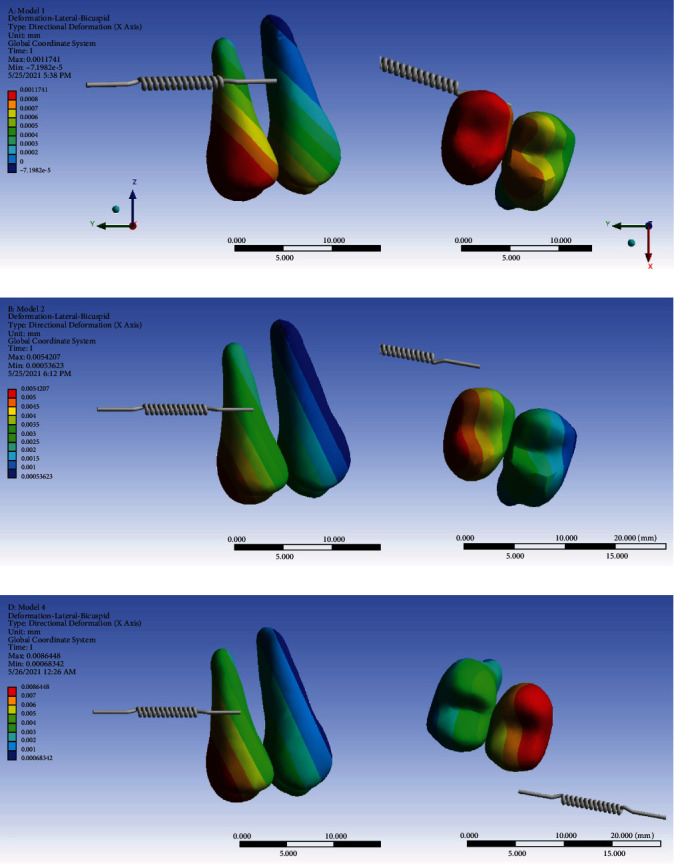
Displacements of the premolars on the *X*-axis (the buccolingual direction) in different models: (a) model 1 with direct anchorage; (b) model 2 with rigid indirect anchorage; (c) model 3 with nonrigid indirect anchorage. Positive values indicate palatalization; negative values indicate buccal movements. The spring shows the force direction.

In all the three models, both the premolar teeth moved in the palatal direction (the positive values on the *X*-axis). This lingualization reduces from the posterior to the anterior segments. Also, it reduces from the coronal tip to the apical area. The extent of palatalization is much less in model 1 compared to the lingualization extents seen in the indirect anchorage models. Between the two indirect anchorage models, the maximum and average lingualizations of model 3 (nonrigid indirect anchorage) were considerably greater than those of model 2 (rigid indirect anchorage), which might imply a higher risk of anchorage loss in model 3 (Figure 17, Table 2).

#### 3.7.2. The Second Molar

**Figure 18:**
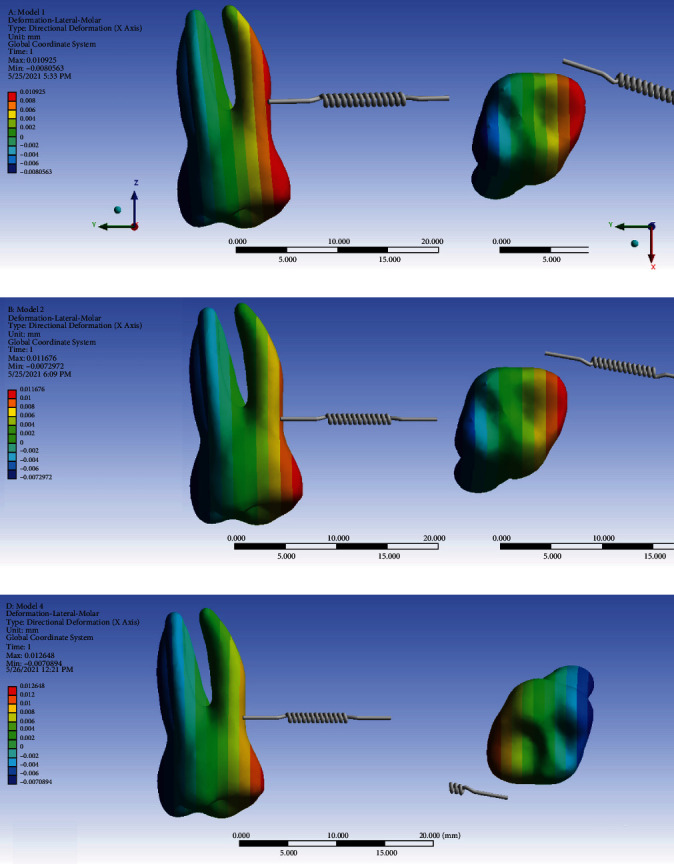
Movements of the second molar in the *X*-axis (the buccolingual direction) in different models: (a) model 1 with direct anchorage; (b) model 2 with rigid indirect anchorage; (c) model 3 with nonrigid indirect anchorage. Positive values indicate lingualization. The spring shows the force direction.

The displacement of the second molar in the *X*-axis was positive (towards the palate) on the mesial side and negative (towards the buccal) on the distal side, indicating a “mesial-in” rotation of the tooth with the axis of rotation almost passing through the long axis of the tooth. The extent of this rotation was quite similar throughout the vertical dimension of the tooth, i.e., from the coronal tip to the root apices. This pattern was observed in all models. And the extents of the maximum and minimum *X*-axis displacements were rather similar among the three models. The average *X*-axis movement was somehow similar in models 1 and 2 (model 1 slightly larger); however, the average *X*-axis displacement was considerably greater in model 3 (Figure 18, Table 2).

### 3.8. Displacement on the *Z*-Axis (Intrusive or Extrusive)

#### 3.8.1. The Premolars

**Figure 19:**
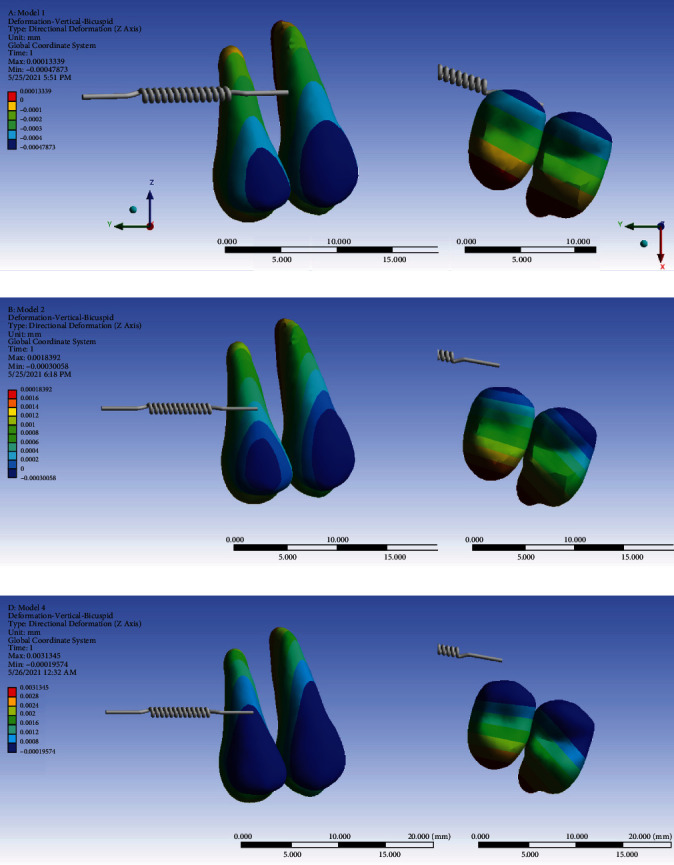
Displacements of the premolars on the *Z*-axis (the intrusive-extrusive direction) in different models: (a) model 1 with direct anchorage; (b) model 2 with rigid indirect anchorage; (c) model 3 with nonrigid indirect anchorage. Positive values indicate intrusion; negative values indicate extrusion. The spring shows the force direction.

The positive values show intrusive motion while negative values show extrusion. In all the models, the buccal side of the crown was extruded while the buccal side of the root was intruded, and this intrusive motion was more vivid towards the apical area. The palatal parts of the crowns and roots underwent intrusive displacement. These indicated a simultaneous buccal root torque and palatal crown tipping, as in uncontrolled tipping. Root and palatal tooth intrusive movements were much greater in the indirect anchorage models (2 and 3) compared to the direct anchorage model (#1). And between the two indirect anchorage models, it was greater in the nonrigid one (model 3). On the other hand, the extrusive movement of the buccal side of the crowns was greater in the direct anchorage model (1) followed by models 2 and 3 (Figure 19, Table 2).

#### 3.8.2. The Second Molar

In model 1, the force is applied close to the center of resistance of the molar; hence, less tipping is created compared to the second and third models. In the second model, the rigid wire holds the base arch in place (in fact, it has inhibited the distortion of the base wire as a result of applying the force). Therefore, less intrusion is observed compared to the third model; in other words, the molar's unwanted movement is reduced.

**Figure 20:**
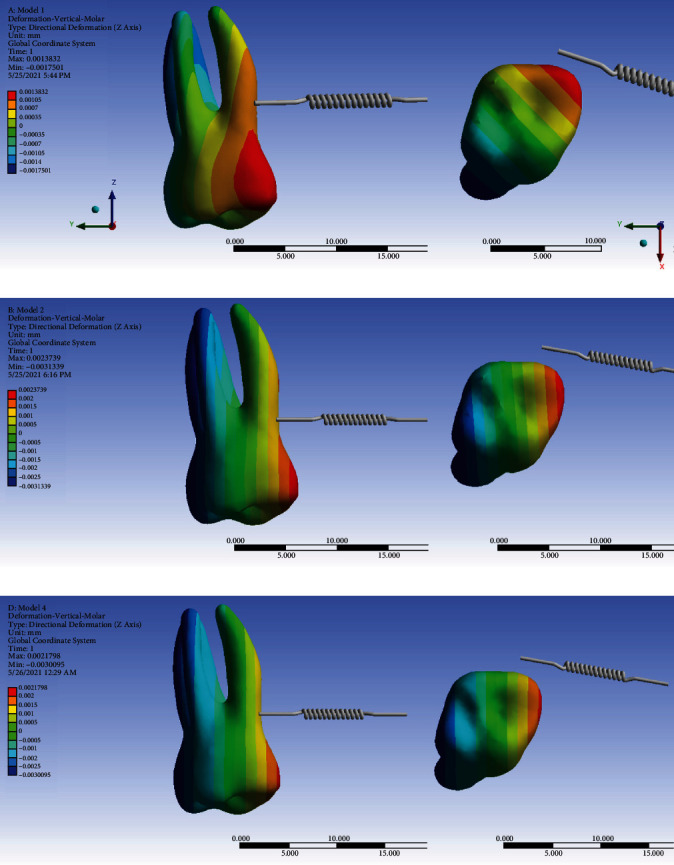
Displacements of the second molar through the *Z*-axis (the intrusive-extrusive direction) in different models: (a) model 1 with direct anchorage; (b) model 2 with rigid indirect anchorage; (c) model 3 with nonrigid indirect anchorage. Negative values indicate extrusion. The spring shows the force direction.

The patterns of *Z*-axis displacements of the second molar differed between the direct anchorage model (model 1) and the two indirect anchorage models (models 2 and 3). In the indirect anchorage models (2 and 3), the mesial side of the tooth (equally from the coronal to the apical areas) tended to have intrusive displacements, while the distal side (again both coronal and radicular areas equally) tended to become extruded; the long axis of the tooth tended to have almost no movement in the *Z*-axis. The maximum intrusive movement was slightly smaller than the maximum extrusive movement, in both models. Overall, the two indirect anchorage models tended to mesially tip the crown of the second molar while at the same time, distally torque its root (both movements around somewhere close to the center of resistance) again causing an uncontrolled tipping (Figure 20, Table 2).

However, the direction of *Z*-axis displacement in the first model differed: instead of the mesial side, the mesiobuccal side (mostly buccal with a small mesial extension) tended to have the maximum intrusive displacement (almost similar for the crown and root), while at the same time, instead of the distal side of the tooth, the distopalatal side (mostly palatal with a small distal extension) of the palatal root followed by the distopalatal side (again mostly palatal with a small distal extension) of the crown had the most extrusive displacement. Again, the long axis had almost no *Z*-axis displacement. The extents of the maximum intrusive and extrusive movements were similar. All of this indicated an uncontrolled tipping with mesiobuccal (more buccal than mesial) tipping of the crown and a distopalatal (mostly palatal) torque of the root around the center of resistance of the tooth (Figure 20, Table 2).

The magnitudes of the maximum intrusion were quite similar between the two indirect anchorage models (2 and 3). They were twice as larger than that in model 1. Similarly, the magnitudes of the maximum extrusions observed were as well similar between models 2 and 3, each being greater than the first model. The “average” *Z*-axis displacements were negative (extrusive) in all the three models; these were very similar in the indirect anchorage models (2 and 3), both being much greater than the rather subtle average (extrusive) movement seen in model 1 (Figure 20, Table 2).

### 3.9. Parametric Assessment of ligature's Elastic Modulus Alterations

**Figure 21:**
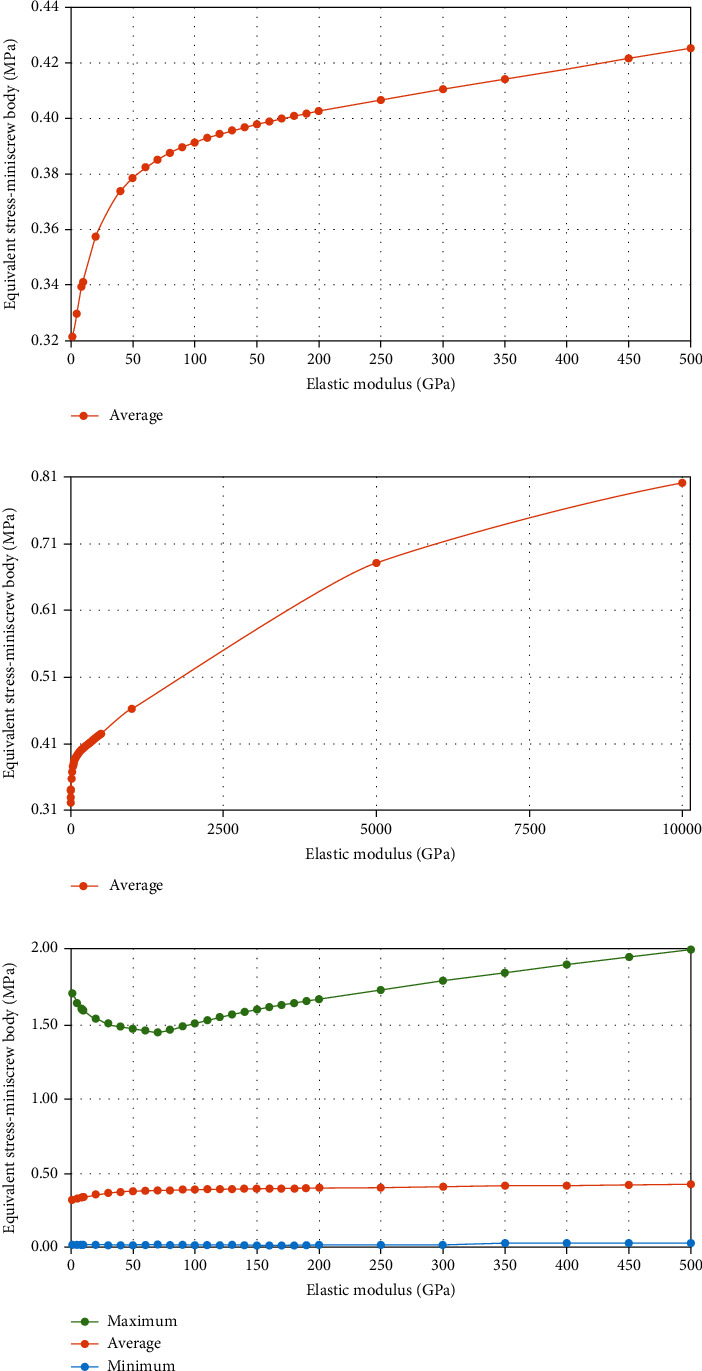
Parametric assessment of the miniscrew stresses (MPa) by increasing the elastic modulus of the ligature wire (GPa). Average changes up to 500 GPa (a); average changes up to 10000 GPa (b); minimum, maximum, and average changes (c).

**Figure 22:**
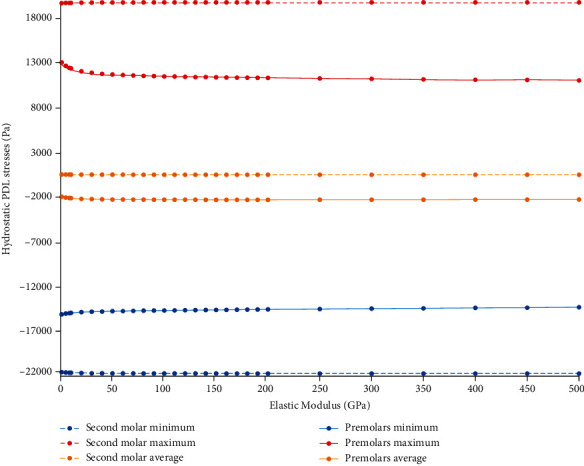
Parametric evaluations of the hydrostatic stresses (Pa) in the PDLs of model 3 at different elastic moduli up to 500 GPa. Negative stresses mean compressive hydrostatic pressure, while positive values mean tensile stress. The minimum stresses (negative values) smaller than -4700 Pa pose an external root resorption risk. Molar stresses are shown using dashed lines. Min: minimum; Max: maximum; Avg: average.

The simulation was repeated with different elastic moduli for the ligature in model 3 (nonrigid indirect anchorage); the effects of such parametric changes on stresses and displacements were assessed. It was shown that the diagram of changes in von Mises stresses would reach a rather steady slope at some elastic moduli (Figure 21, Supplementary Table 1). A similar pattern was observed for the PDL hydrostatic pressures, although with a less remarkable overall change in hydrostatic pressures as a function of increasing the modulus of elasticity. In this regard, the minimum hydrostatic pressure remained below the critical value of -0.0047 MPa (as the threshold for root resorption risk), meaning that there was a risk of root resorption at all different moduli of elasticity (Figure 22, Supplementary Table 2).

#### 3.9.1. Displacements in the *Y*-Axis (Mesiodistal)

**Figure 23:**
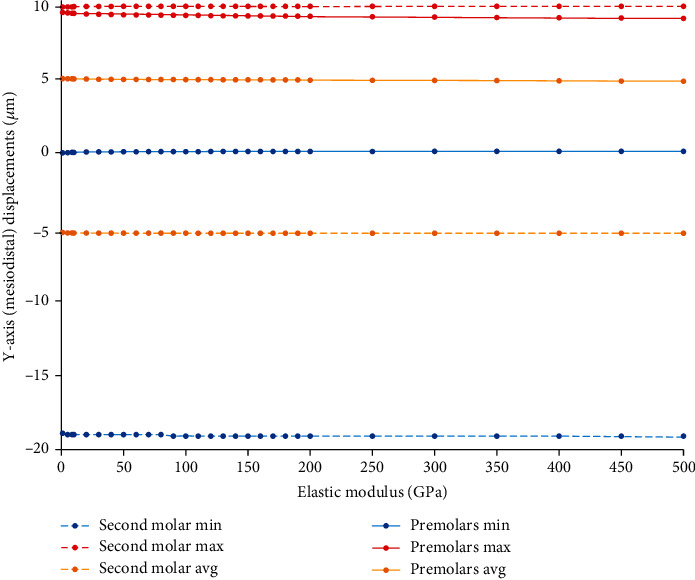
Changes in the extent of *Y*-axis (mesiodistal) displacements of the anchorage and active unit teeth (*μ*m) in model 3 by increasing the elastic modulus of the ligature wire up to 500 GPa. Positive values indicate distalization while negative values indicate mesialization. Molar movements are shown using dashed lines. Min: minimum; Max: maximum; Avg: average.

By increasing the elastic modulus of the wire, the distal movement of the premolars decreased, which means strengthening the anchorage and more resistance to anchorage loss (Figure 23, Supplementary Table 3).

#### 3.9.2. Displacements in the *X*-Axis (Buccolingual)

**Figure 24:**
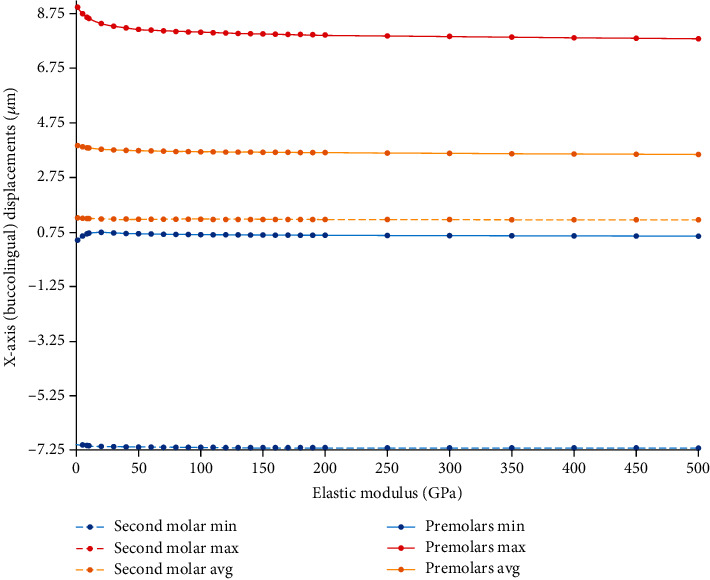
Changes in the extent of *X*-axis (buccolingual) displacements of the anchorage and active unit teeth (*μ*m) in model 3 by increasing the elastic modulus of the ligature wire up to 500 GPa. Negative values indicate buccal movement, while positive values indicate palatalization. Molar movements are shown using dashed lines. Min: minimum; Max: maximum; Avg: average.

With increasing the elastic modulus of the wire, a slight decrease in premolar displacement in the buccolingual axis was observed (Figure 24, Supplementary Table 4). As the rigidity of the wire increases, its resistance to the buccolingual displacement increases and prevents the palatal movement of the teeth.

#### 3.9.3. Movements in the *Z*-Axis (Intrusive/Extrusive)

**Figure 25:**
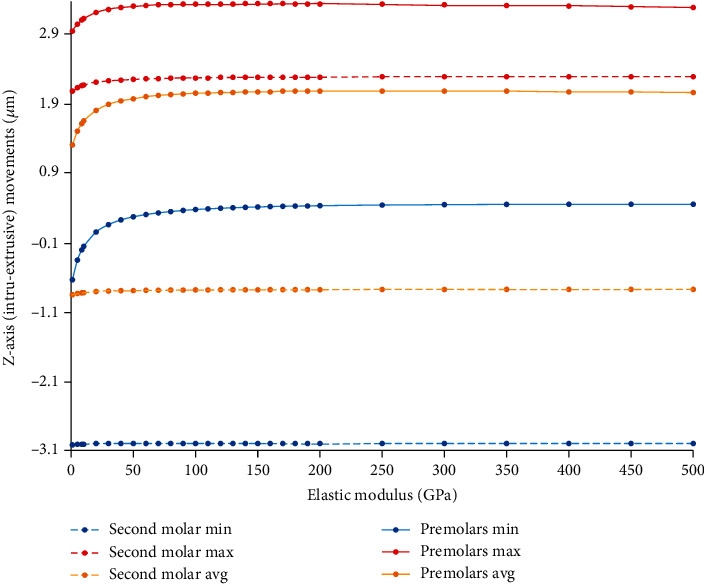
Alterations in the extent of *Z*-axis (intrusive-extrusive) displacements of the teeth (*μ*m) in the nonrigid indirect anchorage model by increasing the elastic modulus of the ligature wire up to 500 GPa. Positive values mean intrusive movement, while negative values mean extrusion. Molar movements are shown using dashed lines. Min: minimum; Max: maximum; Avg: average.

By increasing the rigidity of the ligature wire, a slight increase in the intrusive movement of the premolars in the vertical axis was observed, while the extrusive motion of the molar was reduced (Figure 25, Supplementary Table 5). The force vector from the ligature wire on the premolar tooth is mesioapical. With increasing the rigidity, the intrusive component also increases, and the intrusive movement of the premolar increases.

## 4. Discussion

Effective management of the space of missing posterior teeth is a major challenge in orthodontic treatment. Posterior edentulous spaces are commonly seen in adult maxillary arches, the most common of which is the loss of the first molars due to caries [4]. The greater the amount of tooth displacement, the more difficult it is to control for side effects. In molar protraction, due to the large mesiodistal dimensions of the tooth, even with temporary skeletal anchorages, controlling the transverse, vertical, and horizontal dimensions is not easy [48]. Three models were considered to investigate the stress distribution. The miniscrews simulated in this study were 1.6 mm in diameter and 8 mm long and were placed vertically in the interdental space of the first and second premolars. According to previous studies, the vertical angle of miniscrew placement reduces stress concentration and increases the likelihood of miniscrew stability [49]. In all models of the present study, a force of 150 g was applied according to previous studies [4]. In the direct anchorage model, the force was applied from the miniscrew to the power arm of the second molar. In the indirect anchorage models, direct and indirect forces were applied from the hook of the second premolars to the second molars, and the teeth of the anchorage unit were fixed using a stainless-steel wire and a ligature steel. In the present study, the average second molar displacement in all 3 models was mesially, palatally, and extrusive. In previous studies, it has been mentioned that there is an increase in the possibility of molar tooth extrusion and subsequently creating an anterior open bite, which emphasizes the importance of controlling the vertical dimension [50].

Our findings had some clinical implications. In the direct anchorage method, there is the lowest possibility of anchorage loss and at the same time the highest risk of failure and loosening of the miniscrew. The stress created in the bone around the miniscrew is almost double compared to the indirect model. In situations where it may not be suitable due to certain factors (such as the young age of the patient or in low-density bones such as the maxillary alveolar process), we should provide measures to improve the stability of the miniscrew. For example, we should remove the force from the miniscrew and use indirect anchorages; this reduces the risk of miniscrew failure. Rigid or nonrigid connection can be used for indirect anchorage (in indirect anchorage, the stress in spongy bone was halved). But indirect anchorage increases the amount of anchorage loss. In the nonrigid indirect anchorage model—using ligature wire—compared to the rigid one—using full-size steel wire, the extent of anchorage loss was about 1.5 times greater. But the advantage of using ligature wire was the less stress created in the miniscrew and its surrounding bone: i.e., about 70% stress reduction was observed in the miniscrew body. In the nonrigid method, the least amount of stress was created in the miniscrew and its surrounding bone; this allows us to use a smaller diameter and length of the miniscrew. This can be useful in some cases such as choosing miniscrews in the interdental area where the space is more limited such as between premolars [51]; in less dense bones like the maxilla; and or in the younger patients who have a lower bone density.

Some studies have shown that there is a need for high anchorage control in molar protraction; according to them, with insufficient anchorage, there is a possibility of tipping of the molar and root resorption [52]. In an earlier research [53], in general, placing the miniscrew in the buccal area (due to the passage of the force through the buccal side of the center of resistance) could lead to unwanted expansion, while placing the miniscrew in the palatal area will reduce the width of the arch [53]. However, in the present study, despite the buccal placement of the miniscrew, the average displacement of molars and premolars was palatally. The direction of the force components will determine the direction of displacement. In our study, the miniscrew was placed in the buccal side. However, due to the shape of the arch being narrower at the location of the miniscrew (between the premolars) compared to the location of the second molar, the components of the force will be mesially and lingually; probably because of this, the movement of the tooth had finally become palatally. In other studies, the unwanted side effects of molar protraction were a buccal force, tipping of adjacent teeth, mesial rotation and buccalization of molar teeth, and crossbite [50]. In the study of Marusamy et al. [52], during maxillary molar protraction, the archwire needs to be expanded at each visit to prevent crossbite due to tooth movement to a narrower arch area [52]. In the study of Holberg et al. [30], mandibular molar protraction was compared using dental anchorage and miniscrew. In the use of dental anchorage, high stresses were reported on the anchorage unit tooth, indicating the high potential of the anchorage loss. In their direct anchorage model, the loss of the anchorage (premolar movement) was effectively prevented, but the high stress around the miniscrew could lead to loosening or loss of the miniscrew. The main problem in this model was the mesial rotation of the molar. To offset this movement, it was recommended to apply force from the lingual to the molar tooth [30]. In the current study, the mesial rotation of the molar was seen in all three models, which is due to the application of force to the buccal side of the center of resistance of the molar tooth. The indirect anchorage model might be preferred by many clinicians because it provides freedom in choosing the location of the miniscrew, reduces the risk of damage to the tooth roots, and allows the use of appropriate biomechanics to control the teeth. In the indirect anchorage model, less displacement was observed on the second premolar tooth compared to the dental anchorage and more displacement was observed compared to the direct anchorage [30]. In our study, the highest displacement of the premolars was related to the indirect nonrigid anchorage model, which was seen in the distal and palatal directions; the lowest displacement of the premolars was related to the direct anchorage model, which in the mesiodistal axis was approximately one tenth of the maximum displacement in the nonrigid indirect anchorage model. The average movement of the premolars in the direct anchorage model was less than indirect models, which can be expected owing to the lack of force exerted onto the premolars in the first model. The results of this study were in line with other studies reporting that the rate of anchorage loss was higher in indirect anchorage models [4, 30]. The average movement of the premolars in the rigid indirect anchorage model of this study was less than that in the nonrigid one: due to the larger size and reduced elasticity of the wire connected to the miniscrew and the second premolar tooth, the movement of the teeth of the anchorage unit is more prevented; but the stress on the miniscrew and the surrounding bone increases, which increases the chance of the miniscrew failing. The results of this study showed that the highest amount of stress in the body of the miniscrew was created in the direct anchorage model (10.916 MPa) while the lowest amount of stress was in the indirect anchorage model (3 MPa). Previous studies have also shown that applying force directly to the miniscrew creates excessive force on the bone around the miniscrew and increases the chances of loosening and failure [30, 54]. The results of our study showed that the stress level in the miniscrew and bone in the three models was lower than the yield stress of titanium (692 MPa) and bone (200 MPa) [55]. Therefore, the miniscrew had sufficient strength against the forces in three models.

Root resorption may occur during orthodontic treatment in the same areas where physiological root resorption begins as these areas are more sensitive to local changes [56]. In fact, orthodontic forces applied to the teeth cause stress distribution in the PDL. PDLs containing very small blood vessels are exposed to this stress. If the pressure is beyond capillary blood pressure, it causes collapse and dysfunction of blood vessels in supplying the blood [57]. According to Schwarz, there is a possibility of root resorption if pressure exceeds capillary blood pressure in the PDL [57–59]. Capillary blood pressure range has been reported between 15 and 35 mm of Hg (0.0020 to 0.0047 MPa) [46, 57, 60]. Mechanical stress can cause changes in blood flow, which is a factor in root resorption [61]. Therefore, hydrostatic pressure can be considered a key factor in assessing the risk of root resorption during orthodontic treatment [57]. The extent of the resorption also depends on the amount of force and torque applied [61–63]. In a recent study, no clear resorption was found in the traction region, which can suggest that odontoclasts do not respond to traction stimuli [64]. The pressure exerted by the tooth root on the bone and the surrounding PDL is the main factor determining the rate of tooth movement, not the force exerted on the tooth crown [65]. The optimal range of stress and force to induce the optimal rate of tooth movement should be between 0.015-0.026 N/mm^2^ and 150-260 grams, and more than this amount will reduce tooth movement [65]. Therefore, the force used in this study was within the optimal range [65]. In all models of this study, this force caused the maximum compressive hydrostatic pressure points (with a value greater than 0.0047 MPa) in the PDL of the second molar, in the mesial sides of its buccal roots (especially over the coronal thirds) and its root trunk as well as the distal side of its palatal root (particularly at the middle and apical thirds). In the nonrigid and rigid indirect anchorage models (models 2 and 3), the highest compressive hydrostatic stress in the PDL was created in the distopalatal root of the premolars and its values were 0.0149 and 0.00859 MPa, respectively. If the compressive hydrostatic stress is greater than 0.0047 MPa, the risk of root resorption is largely increased [46, 47]. Therefore, in the nonrigid model, we expect more resorption in the teeth of the anchorage unit due to the greater movement and compression of these teeth against the bone. In the direct anchorage model, the maximum compressive hydrostatic stress was 0.0015 MPa in the premolars, which was less than the resorption threshold and well tolerated.

In the study of Nihara et al. [66], to determine the most desirable force system for the protraction of mandibular molars using a miniscrew in the interradicular area in the buccal side of the mandible, a power arm was used on the molars at different lengths (2 to 10 mm). The position of the miniscrew in different models was placed up to 8 mm more apical than the level of the gingival margin of the second molar (with vertical intervals of 2 mm). They concluded that mesiodistal tipping decreased with increasing the power arm length to 8 mm; at the 10 mm length, distal crown tipping occurred despite the mesial force, but the buccolingual displacement changed less and remained a buccal tipping [66]. In the direct anchorage model of our study, using a power arm, the force was applied near the center of resistance. In this model, the average and maximum movement in the mesial direction were less than other models in which the force was applied farther from the center of resistance of the molar.

In our study, the application of 150 g force in the rigid indirect anchorage model resulted in 0.129 MPa of stress in the spongy bone, and in the nonrigid indirect anchorage model resulted in 0.107 MPa of stress in the spongy bone; this higher stress in the rigid model can play a role in miniscrew failure. It was found that factors such as the connection of ligature wire or elastomeric chain increase the risk of miniscrew failure through plaque accumulation: plaque builds up around the elastomeric chain, leading to more inflammation around the miniscrew and more failure [67].

Besides root resorption, there is bone resorption risk as well. According to previous studies, increasing the level of stress and pressure can disrupt periosteal blood supply and lead to necrosis and bone resorption [68–70]. In this study, we preferred to calculate bone stresses rather than deformities. Since the model is considered having a linear elastic behavior, there is total reciprocity between strain (deformity) and stress; in other words, any strain level (that causes microdamage or other failure types caused by deformation) can be replaced with a corresponding stress level. Thus, we felt it would be more appropriate to calculate stresses (instead of deformities) for the sake of greater simplicity and comprehensibility of the outcomes and their interpretations. Moreover, there was no “stress or strain threshold” for the objective calculation of cancellous bone resorption in the literature. Therefore, we had to stick with the PDL pressure which has an objective threshold for root resorption and hence is examinable scientifically and objectively.

SS ligatures are very soft and malleable wires made from deadsoft wires [14]. In previous studies, different values were reported for the elastic modulus of this wire: it was reported 8500 MPa, 130000 MPa for 0.007-inch diameter, and 140000 MPa for 0.011-inch diameter [34]. In various FEA studies, different moduli of elasticity had been used, such as 160 gigapascals [35], 168 gigapascals [36], 176 gigapascals [37], 180 gigapascals [38], and 200 gigapascals [39, 40]. In the present study, with increasing ligature rigidity, the palatal and distal movements of the premolars decreased and their intrusive movement increased. In other words, with the increase in rigidity of the ligature wire, the resistance to anchorage loss was increased. The increase in the premolar intrusive displacement might seem to be caused due to the apical direction of the connection of the anchorage teeth to the miniscrew. As wire rigidity increased, the extrusion and palatal movement of the molar decreased as well, which might be related to the increased resistance of the ligature wire to movements. Molar mesialization was slightly enhanced by increasing the wire rigidity. The increase in the ligature wire rigidity might not much reduce the risk of root resorption. No similar study was available to compare our results with.

In the first model, the distal movement of the root of the second premolar could be seen; it was very small and was caused by the spread of force and the stretching of the gingival fibers and hence not posing any serious anchorage loss risk. This distal movement was much smaller than the mesial movement of the root of the second molar. In the buccolingual axis (*X*-axis), the palatal displacement of the mesial half of the root of the second molar and the buccal displacement of the distal half of the root of the second molar can be seen. In fact, the rotation of the tooth took place around the vertical axis. But in the second premolar region, the palatal displacement of the root of the second premolar is observed, and the magnitude of this displacement is reduced towards the apical side. The amount of molar movement is more than the premolar. It seems that because the force is applied directly to the second molar, the greatest movement should be expected in it. In the vertical axis (*Z*-axis), an extrusive movement can be seen in the region of the root of the second premolar, which decreases toward the apical side. In the mesiobuccal root of the second molar, the movement is intrusive, while in the distobuccal and palatal roots of the second molar, the movement is extrusive. The average displacement of the second molar is greater than the second premolar.

In the second model, the distal displacement of the second premolar root and the mesial displacement of the molar root can be seen in the *Y*-axis. In both teeth, the magnitude of this displacement decreases towards the apical. It should be noted that the amount of molar root movement is greater than the amount of second premolar root movement. In this model, the lingual displacement of the second premolar root and the lingual displacement of the molar's mesiobuccal root and the buccal displacement of its distobuccal and palatal roots can be seen in the *X*-axis. The lingual displacement of the second premolar is less than that of the second molar, which seems reasonable considering the existence of a full-sized rigid wire that prevents the movement of the premolar. In the *Z*-axis, the intrusive movement of the premolar roots can be seen, which increases toward the apical end. In the second molar region, in the mesiobuccal root, the movement is mainly intrusive, and in the distobuccal and palatal roots, the movement is mainly extrusive. The amount of displacement of the molar root is greater than that of the premolar, which is due to the restraint caused by the rigid connecting wire between the miniscrew and the second premolar tooth.

In the third model, in the *Y*-axis, these movements were noted: the distal displacement of the root of the second premolar (toward the apical side, the magnitude of this displacement decreases); the mesial displacement of the buccal roots of the second molar; and the distal displacement of the palatal root of the second molar. In fact, the rotation of the second molar is observed around the vertical axis. Molar root movement is greater than premolar root movement. In *X*- and *Z*-axes, the displacement pattern observed is similar to the rigid model.

Hence, overall, it can be said that in direct anchorage, the highest amount of root displacement is observed in the second molar roots; in the premolars, the amount of displacement is very small, practically without anchorage loss. The total displacement of the root of the second premolar is distopalatal and extrusive, while the second molar roots are rotated around the vertical axis (i.e., the buccal roots were mesialized and the palatal root was distalized); furthermore, the mesiobuccal part of the second molar was intruded, while distobuccal and palatal parts were extrusion. In rigid anchorage and nonrigid anchorage, it is again observed that the root displacement of the second molar is greater than that of the premolar. In these two types of anchorage, the types of movements observed in the root area were similar to direct anchorage; with the difference that in premolar roots, the movement is intrusive (which seems to be due to the presence of a connecting wire between the tooth and the miniscrew).

One of the limitations of this finite element study is the simplification of modeling the complex tissues and structures. For example, bone properties are assumed to be isotropic and time-independent linear elastic, which differs from bone behavior in the clinic. In addition, FEA disregards numerous parameters such as various patients' sexes, ages, statures, or genetics. Anatomy and structural properties vary from person to person. This examination is essentially a static analysis which is hard to generalize to clinical situations; hence, its implementation and interpretation need cautious decision-making [71]. Furthermore, FEA may not simulate long-term kinetics of tooth movement, needing slow and quite complicated biological alterations in live tissues like remodeling of the PDL and bone [72]. Thus, future clinical studies are needed to assess our results. However, the finite element method is advantageous over clinical or even in vitro studies, as it can provide a very precise and detailed overview of the mechanics of the whole system and each of its parts, not possible with any other approach. Applying biologically comprehensible and multifaceted in silico simulations may allow the prediction of root resorption risk and also the clarification of some mechanisms underlying orthodontic tooth movement [73].

## 5. Conclusions

Within the limitations of this FEA simulation, the following could be concluded:

1. The miniscrew stress and the spongy bone stress were much larger in the direct anchorage compared to the indirect anchorage models
2. The lowest miniscrew and cancellous bone stresses were seen in the nonrigid indirect anchorage model
3. Palatalization of the premolars in the nonrigid indirect anchorage model was considerably greater than the rigid one. Between the indirect anchorage models, the nonrigid anchorage model had a greater root intrusion
4. In the indirect models, there might be some risk of root resorption of the anchorage teeth, and this might be greater in the nonrigid indirect anchorage compared to the rigid one. In the direct model, the greatest compressive and tensile hydrostatic stresses were observed in the PDL parts around the miniscrew
5. Hydrostatic pressure patterns of the molar PDLs might be rather similar in the three tested models, with the tensile stress concentrating in the distal sides of the mesiobuccal and distobuccal roots and the mesial side of the palatal root, and compressive hydrostatic pressures seen on the mesial sides of the buccal roots, on the mesial side of the root trunk, and on the distal side of the palatal root. The compressive stresses were the lowest and highest in the direct anchorage and rigid indirect anchorage models, respectively; however, extents of tensile stresses were similar
6. The miniscrew load of 150 g might not break the titanium body of the implant in any of the three models. However, the miniscrew and bone stresses imply a higher risk of miniscrew loosening in the direct anchorage model and a lower one in the nonrigid indirect anchorage method. Therefore, it seems that when the bone quality around the mini-implant is not appropriate, shifting to the nonrigid indirect anchorage paradigm might be preferable to avoid a high risk of miniscrew failure
7. Using ligature wire increases the risk of anchorage loss and root resorption in the anchorage unit
8. Between the two indirect anchorage methods, the rigid one might provide a greater extent or rate of molar protraction
9. The direct anchorage method is more likely to provide a bodily (but slower maximum) movement of the molar compared to the indirect anchorage methods
10. Increasing the rigidity of the connecting wire in the nonrigid indirect anchorage method might slightly accelerate the mesialization of the second molar, while reducing the risk of anchorage loss

## Figures and Tables

**Table 1:** Material properties.

Material	Elastic modulus (MPa)	Poisson's ratio
Cortical bone [41]	1000	0.3
Cancellous bone [41]	500	0.3
Dentine [41]	18600	0.3
PDL [42]	0.15	0.45
Stainless steel [43]	200000	0.3
Miniscrew titanium G5 [44]	115000	0.33
Ligature (dead soft wire) [45]	8500	0.3

**Table 2:** Simulation results.

Property	Scope	Model 1			Model 2			Model 3		
		Minimum	Maximum	Average	Minimum	Maximum	Average	Minimum	Maximum	Average
Von Mises stress (Pa)	All bodies	0.034133	96851000	131320	0.19869	51224000	171090	0.19947	46540000	265410
	Miniscrew	84.003	10916000	1813800	136130	32146000	2090200	3188.4	20948000	314710
	MC				36528	33983000	2702400	2129.3	39577000	1191800
	CB	1.3817	214350	8259.4	7.7935	129060	8715.1	6.921	107600	7201.3

Hydrostatic stress (Pa) of PDL	All teeth	-18550	19086	41.912	-22380	20448	-41.21	-21616	19891	-41.715
	Molar	-18550	19086	256.85	-22380	20448	430.59	-21616	19891	615.05
	Bicuspid	-1593.4	3491.4	-139.38	-8594.6	7366	-1064.7	-14919	12599	-2008.1

Displacement-Y (*μ*m)	All bodies	-29.86	7.8788	-0.27785	-22.776	9.2276	-0.16363	-23.173	12.898	0.58086
	All teeth	-15.046	7.8788	-0.76672	-18.853	9.2276	-0.46188	-18.982	9.5846	-0.35065
	Molar	-15.046	7.8788	-4.5531	-18.853	9.2276	-5.6413	-18.982	9.5846	-5.5237
	Bicuspid	-0.047037	0.93044	0.41375	0.023664	5.7812	3.0526	-0.14605	9.1463	4.7655

Displacement-X (*μ*m)	Molar	-8.0563	10.925	0.85247	-7.2972	11.676	0.76299	-7.0894	12.648	1.2485
	Bicuspid	-0.071982	1.1741	0.54464	0.53623	5.4207	2.5706	0.68342	8.6448	3.8454

Displacement-Z (*μ*m)	Molar	-1.7501	1.3832	-0.19644	-3.1339	2.3739	-0.86759	-3.0095	2.1798	-0.81859
	Bicuspid	-0.47873	0.13339	-0.12516	-0.30058	1.8392	0.93506	-0.19574	3.1345	1.6291

## Data Availability

All data are presented as the article tables and figures.

## Supplementary Materials

Supplementary Materials Supplementary Tables 1 to 5 present the details of parametric simulations.
