# Supplementary material for: Effects of Rigid and Nonrigid Connections between the Miniscrew and Anchorage Tooth on Dynamics, Efficacy, and Adverse Effects of Maxillary Second Molar Protraction: A Finite Element Analysis
Source: Biomed Res Int. 2022 Oct 14;2022:4714347. doi: 10.1155/2022/4714347 (PMC9586811; doi:10.1155/2022/4714347)
Supplement: Supplementary Materials — Supplementary Tables 1 to 5 present the details of parametric simulations. [file 4714347.f1.docx]

Supplementary Table 1**.** Parametric assessments of the equivalent stresses (Pa) in the model 3 (non-rigid indirect anchorage) per different elastic moduli (GPa).

| **Elastic Modulus (GPa)** | **Miniscrew body** | | | **Cortical bone** | | | **Cancellous bone** | | |
| --- | --- | --- | --- | --- | --- | --- | --- | --- | --- |
|  | **Min** | **Max** | **Avg** | **Min** | **Max** | **Avg** | **Min** | **Max** | **Avg** |
| 1 | 18300 | 1710000 | 321000 | 3.9 | 221000 | 10700 | 5.62 | 92700 | 7200 |
| 5 | 17300 | 1650000 | 330000 | 3.38 | 243000 | 10900 | 7.01 | 101000 | 7200 |
| 8.5 | 19900 | 1610000 | 340000 | 3.46 | 257000 | 11000 | 6.92 | 108000 | 7200 |
| 10 | 17900 | 1600000 | 341000 | 3.55 | 262000 | 11000 | 6.91 | 110000 | 7200 |
| 20 | 18500 | 1540000 | 357000 | 4.2 | 285000 | 11300 | 6.94 | 120000 | 7220 |
| 30 | 15400 | 1510000 | 367000 | 4.69 | 299000 | 11400 | 6.87 | 127000 | 7230 |
| 40 | 15300 | 1490000 | 374000 | 5.02 | 307000 | 11500 | 6.45 | 130000 | 7230 |
| 50 | 16500 | 1480000 | 379000 | 5.26 | 313000 | 11600 | 6.18 | 133000 | 7240 |
| 60 | 18200 | 1460000 | 382000 | 5.43 | 317000 | 11700 | 6 | 135000 | 7240 |
| 70 | 20100 | 1450000 | 385000 | 5.57 | 320000 | 11700 | 5.88 | 137000 | 7250 |
| 80 | 15100 | 1470000 | 388000 | 5.68 | 323000 | 11800 | 5.79 | 138000 | 7250 |
| 90 | 18500 | 1490000 | 390000 | 5.76 | 325000 | 11800 | 5.72 | 139000 | 7250 |
| 100 | 15200 | 1510000 | 391000 | 5.84 | 326000 | 11800 | 5.67 | 140000 | 7250 |
| 110 | 14700 | 1530000 | 393000 | 5.9 | 327000 | 11800 | 5.63 | 141000 | 7260 |
| 120 | 16400 | 1550000 | 394000 | 5.95 | 328000 | 11900 | 5.59 | 142000 | 7260 |
| 130 | 18700 | 1570000 | 396000 | 5.99 | 329000 | 11900 | 5.57 | 142000 | 7260 |
| 140 | 15600 | 1590000 | 397000 | 6.03 | 330000 | 11900 | 5.55 | 143000 | 7260 |
| 150 | 13500 | 1610000 | 398000 | 6.06 | 331000 | 11900 | 5.53 | 143000 | 7260 |
| 160 | 12600 | 1620000 | 399000 | 6.09 | 331000 | 11900 | 5.51 | 144000 | 7260 |
| 170 | 13000 | 1640000 | 400000 | 6.11 | 332000 | 12000 | 5.5 | 144000 | 7270 |
| 180 | 14300 | 1650000 | 401000 | 6.14 | 332000 | 12000 | 5.49 | 144000 | 7270 |
| 190 | 16200 | 1660000 | 402000 | 6.16 | 333000 | 12000 | 5.48 | 145000 | 7270 |
| 200 | 18600 | 1680000 | 403000 | 6.17 | 333000 | 12000 | 5.48 | 145000 | 7270 |
| 250 | 18100 | 1740000 | 407000 | 6.24 | 334000 | 12100 | 5.46 | 146000 | 7280 |
| 300 | 17200 | 1800000 | 411000 | 6.21 | 335000 | 12100 | 5.46 | 147000 | 7280 |
| 350 | 30200 | 1850000 | 414000 | 6.18 | 335000 | 12200 | 5.47 | 148000 | 7290 |
| 400 | 30600 | 1910000 | 418000 | 6.15 | 335000 | 12200 | 5.49 | 149000 | 7290 |
| 450 | 29400 | 1960000 | 422000 | 6.14 | 335000 | 12300 | 5.51 | 149000 | 7300 |
| 500 | 29500 | 2010000 | 425000 | 6.12 | 335000 | 12300 | 5.53 | 150000 | 7310 |
| 1000 | 19400 | 2440000 | 462000 | 6.03 | 336000 | 12800 | 5.8 | 155000 | 7380 |
| 5000 | 47700 | 4460000 | 681000 | 5.52 | 527000 | 14900 | 7.01 | 189000 | 7830 |
| 10000 | 61900 | 5430000 | 802000 | 4.92 | 641000 | 16000 | 6.72 | 215000 | 8090 |

**Min**, minimum; **Max**, maximum; **Avg**, average.

Supplementary Table 2**.** Parametric evaluations of the hydrostatic stresses (Pa) in the PDLs of the model 3 at different elastic moduli (GPa). Negative stresses mean compressive hydrostatic pressure, while positive values mean tensile stress. The minimum stresses (negative values) smaller than -4700 Pa pose an external root resorption risk.

| **Elastic Modulus (GPa)** | **Second Molar** | | | **Premolars** | | |
| --- | --- | --- | --- | --- | --- | --- |
|  | **Minimum** | **Maximum** | **Average** | **Minimum** | **Maximum** | **Average** |
| 1 | -21560 | 19860 | 620 | -15070 | 13200 | -1870 |
| 5 | -21590 | 19880 | 620 | -14970 | 12820 | -1960 |
| 8.5 | -21620 | 19890 | 620 | -14920 | 12600 | -2010 |
| 10 | -21620 | 19900 | 610 | -14900 | 12530 | -2020 |
| 20 | -21650 | 19910 | 610 | -14820 | 12210 | -2100 |
| 30 | -21670 | 19920 | 610 | -14770 | 12040 | -2130 |
| 40 | -21680 | 19930 | 610 | -14740 | 11930 | -2150 |
| 50 | -21690 | 19930 | 610 | -14710 | 11850 | -2170 |
| 60 | -21690 | 19930 | 610 | -14690 | 11790 | -2180 |
| 70 | -21690 | 19930 | 600 | -14670 | 11740 | -2190 |
| 80 | -21700 | 19940 | 600 | -14650 | 11700 | -2190 |
| 90 | -21700 | 19940 | 600 | -14640 | 11670 | -2200 |
| 100 | -21700 | 19940 | 600 | -14620 | 11640 | -2200 |
| 110 | -21700 | 19940 | 600 | -14610 | 11620 | -2200 |
| 120 | -21700 | 19940 | 600 | -14600 | 11590 | -2200 |
| 130 | -21700 | 19940 | 600 | -14590 | 11570 | -2200 |
| 140 | -21700 | 19940 | 600 | -14580 | 11560 | -2210 |
| 150 | -21700 | 19940 | 600 | -14560 | 11540 | -2210 |
| 160 | -21710 | 19940 | 600 | -14550 | 11520 | -2210 |
| 170 | -21710 | 19940 | 600 | -14540 | 11510 | -2210 |
| 180 | -21710 | 19940 | 600 | -14530 | 11490 | -2210 |
| 190 | -21710 | 19940 | 600 | -14520 | 11480 | -2210 |
| 200 | -21710 | 19940 | 600 | -14510 | 11470 | -2210 |
| 250 | -21710 | 19940 | 600 | -14470 | 11410 | -2200 |
| 300 | -21710 | 19940 | 600 | -14420 | 11360 | -2200 |
| 350 | -21710 | 19940 | 600 | -14380 | 11320 | -2200 |
| 400 | -21710 | 19940 | 600 | -14340 | 11270 | -2190 |
| 450 | -21710 | 19940 | 600 | -14310 | 11240 | -2190 |
| 500 | -21710 | 19940 | 600 | -14270 | 11200 | -2180 |
| 1000 | -21700 | 19930 | 610 | -13960 | 10910 | -2130 |
| 5000 | -21670 | 19890 | 610 | -12680 | 9750 | -1900 |
| 10000 | -21650 | 19880 | 620 | -12030 | 9160 | -1780 |

Supplementary Table 3**.** Changes in the extent of **Y-axis (mesiodistal)** displacements of the anchorage and active unit teeth (µm) in the model 3 by increasing the elastic modulus of the ligature wire (GPa). Positive values indicate distalization while negative values indicate mesialization.

| **Elastic Modulus (GPa)** | **Second Molar** | | | **Premolars** | | |
| --- | --- | --- | --- | --- | --- | --- |
|  | **Min** | **Max** | **Avg** | **Min** | **Max** | **Avg** |
| 1 | -18.9 | 9.57 | -5.51 | -0.182 | 9.21 | 4.78 |
| 5 | -19 | 9.58 | -5.52 | -0.159 | 9.17 | 4.77 |
| 8.5 | -19 | 9.58 | -5.52 | -0.146 | 9.15 | 4.77 |
| 10 | -19 | 9.59 | -5.53 | -0.142 | 9.14 | 4.76 |
| 20 | -19 | 9.6 | -5.53 | -0.123 | 9.1 | 4.75 |
| 30 | -19 | 9.6 | -5.54 | -0.113 | 9.08 | 4.74 |
| 40 | -19 | 9.6 | -5.54 | -0.107 | 9.06 | 4.74 |
| 50 | -19 | 9.61 | -5.54 | -0.102 | 9.05 | 4.73 |
| 60 | -19 | 9.61 | -5.54 | -0.0991 | 9.04 | 4.73 |
| 70 | -19 | 9.61 | -5.54 | -0.0966 | 9.03 | 4.72 |
| 80 | -19 | 9.61 | -5.54 | -0.0947 | 9.02 | 4.72 |
| 90 | -19.1 | 9.61 | -5.54 | -0.093 | 9.01 | 4.72 |
| 100 | -19.1 | 9.61 | -5.54 | -0.0917 | 9 | 4.71 |
| 110 | -19.1 | 9.61 | -5.55 | -0.0905 | 9 | 4.71 |
| 120 | -19.1 | 9.61 | -5.55 | -0.0895 | 8.99 | 4.71 |
| 130 | -19.1 | 9.61 | -5.55 | -0.0886 | 8.98 | 4.7 |
| 140 | -19.1 | 9.61 | -5.55 | -0.0879 | 8.98 | 4.7 |
| 150 | -19.1 | 9.61 | -5.55 | -0.0872 | 8.97 | 4.7 |
| 160 | -19.1 | 9.61 | -5.55 | -0.0865 | 8.97 | 4.7 |
| 170 | -19.1 | 9.61 | -5.55 | -0.086 | 8.96 | 4.69 |
| 180 | -19.1 | 9.61 | -5.55 | -0.0854 | 8.96 | 4.69 |
| 190 | -19.1 | 9.61 | -5.55 | -0.0849 | 8.95 | 4.69 |
| 200 | -19.1 | 9.61 | -5.55 | -0.0845 | 8.94 | 4.68 |
| 250 | -19.1 | 9.61 | -5.55 | -0.0826 | 8.92 | 4.67 |
| 300 | -19.1 | 9.61 | -5.55 | -0.0812 | 8.89 | 4.66 |
| 350 | -19.1 | 9.61 | -5.55 | -0.0801 | 8.87 | 4.65 |
| 400 | -19.1 | 9.61 | -5.55 | -0.0792 | 8.85 | 4.63 |
| 450 | -19.1 | 9.61 | -5.55 | -0.0783 | 8.83 | 4.62 |
| 500 | -19.1 | 9.61 | -5.55 | -0.0776 | 8.81 | 4.61 |
| 1000 | -19 | 9.6 | -5.55 | -0.073 | 8.63 | 4.52 |
| 5000 | -19 | 9.57 | -5.54 | -0.0686 | 7.92 | 4.12 |
| 10000 | -19 | 9.55 | -5.54 | -0.0788 | 7.57 | 3.92 |

**Min**, minimum; **Max**, maximum; **Avg**, average.

Supplementary Table 4**.** Changes in the amount of X-axis (buccolingual) displacements of the teeth (µm) of the 3rd model by increasing the elastic modulus of the ligature wire (GPa). Negative values indicate buccal movement, while positive values indicate palatalization.

| **Elastic Modulus (GPa)** | **Second Molar** | | | **Premolars** | | |
| --- | --- | --- | --- | --- | --- | --- |
|  | **Min** | **Max** | **Avg** | **Min** | **Max** | **Avg** |
| 1 | -7.04 | 12.7 | 1.27 | 0.455 | 9.01 | 3.93 |
| 5 | -7.07 | 12.7 | 1.26 | 0.602 | 8.77 | 3.88 |
| 8.5 | -7.09 | 12.6 | 1.25 | 0.683 | 8.64 | 3.85 |
| 10 | -7.1 | 12.6 | 1.25 | 0.71 | 8.6 | 3.84 |
| 20 | -7.12 | 12.6 | 1.23 | 0.74 | 8.41 | 3.79 |
| 30 | -7.13 | 12.6 | 1.23 | 0.716 | 8.31 | 3.77 |
| 40 | -7.14 | 12.6 | 1.23 | 0.699 | 8.25 | 3.75 |
| 50 | -7.15 | 12.6 | 1.22 | 0.687 | 8.2 | 3.74 |
| 60 | -7.15 | 12.6 | 1.22 | 0.678 | 8.17 | 3.73 |
| 70 | -7.16 | 12.5 | 1.22 | 0.672 | 8.14 | 3.72 |
| 80 | -7.16 | 12.5 | 1.22 | 0.666 | 8.12 | 3.71 |
| 90 | -7.16 | 12.5 | 1.22 | 0.661 | 8.1 | 3.71 |
| 100 | -7.16 | 12.5 | 1.22 | 0.657 | 8.09 | 3.7 |
| 110 | -7.16 | 12.5 | 1.21 | 0.652 | 8.07 | 3.7 |
| 120 | -7.17 | 12.5 | 1.21 | 0.649 | 8.06 | 3.69 |
| 130 | -7.17 | 12.5 | 1.21 | 0.646 | 8.05 | 3.69 |
| 140 | -7.17 | 12.5 | 1.21 | 0.643 | 8.04 | 3.69 |
| 150 | -7.17 | 12.5 | 1.21 | 0.64 | 8.03 | 3.68 |
| 160 | -7.17 | 12.5 | 1.21 | 0.638 | 8.02 | 3.68 |
| 170 | -7.17 | 12.5 | 1.21 | 0.636 | 8.01 | 3.68 |
| 180 | -7.17 | 12.5 | 1.21 | 0.634 | 8.01 | 3.67 |
| 190 | -7.17 | 12.5 | 1.21 | 0.632 | 8 | 3.67 |
| 200 | -7.17 | 12.5 | 1.21 | 0.63 | 7.99 | 3.67 |
| 250 | -7.18 | 12.5 | 1.21 | 0.623 | 7.96 | 3.65 |
| 300 | -7.18 | 12.5 | 1.21 | 0.617 | 7.94 | 3.64 |
| 350 | -7.18 | 12.5 | 1.2 | 0.613 | 7.91 | 3.63 |
| 400 | -7.18 | 12.5 | 1.2 | 0.609 | 7.89 | 3.62 |
| 450 | -7.18 | 12.5 | 1.2 | 0.605 | 7.87 | 3.61 |
| 500 | -7.19 | 12.5 | 1.2 | 0.601 | 7.85 | 3.6 |
| 1000 | -7.2 | 12.5 | 1.19 | 0.576 | 7.7 | 3.52 |
| 5000 | -7.24 | 12.4 | 1.15 | 0.489 | 7.13 | 3.19 |
| 10000 | -7.26 | 12.3 | 1.14 | 0.447 | 6.84 | 3.02 |

**Min**, minimum; **Max**, maximum; **Avg**, average.

Supplementary Table 5**.** Alterations in the extent of Z-axis (intrusive-extrusive) displacements of the teeth (µm) in the non-rigid indirect anchorage model by increasing the elastic modulus of the ligature wire (GPa). Positive values mean intrusive movement, while negative values mean extrusion.

| **Elastic Modulus (GPa)** | **Second Molar** | | | **Premolars** | | |
| --- | --- | --- | --- | --- | --- | --- |
|  | **Min** | **Max** | **Avg** | **Min** | **Max** | **Avg** |
| 1 | -3.02 | 2.1 | -0.847 | -0.63 | 2.97 | 1.32 |
| 5 | -3.01 | 2.15 | -0.829 | -0.345 | 3.07 | 1.52 |
| 8.5 | -3.01 | 2.18 | -0.819 | -0.196 | 3.13 | 1.63 |
| 10 | -3.01 | 2.19 | -0.815 | -0.147 | 3.15 | 1.67 |
| 20 | -3 | 2.23 | -0.801 | 0.0608 | 3.24 | 1.82 |
| 30 | -3 | 2.25 | -0.793 | 0.169 | 3.28 | 1.91 |
| 40 | -3 | 2.26 | -0.788 | 0.236 | 3.31 | 1.96 |
| 50 | -3 | 2.27 | -0.785 | 0.281 | 3.33 | 1.99 |
| 60 | -3 | 2.28 | -0.782 | 0.314 | 3.34 | 2.02 |
| 70 | -3 | 2.28 | -0.78 | 0.339 | 3.35 | 2.04 |
| 80 | -3 | 2.29 | -0.779 | 0.358 | 3.35 | 2.05 |
| 90 | -3 | 2.29 | -0.778 | 0.373 | 3.36 | 2.06 |
| 100 | -3 | 2.29 | -0.777 | 0.386 | 3.36 | 2.07 |
| 110 | -3 | 2.29 | -0.776 | 0.396 | 3.36 | 2.07 |
| 120 | -3 | 2.3 | -0.775 | 0.405 | 3.36 | 2.08 |
| 130 | -3 | 2.3 | -0.775 | 0.412 | 3.36 | 2.08 |
| 140 | -3 | 2.3 | -0.774 | 0.418 | 3.37 | 2.09 |
| 150 | -3 | 2.3 | -0.774 | 0.424 | 3.37 | 2.09 |
| 160 | -3 | 2.3 | -0.773 | 0.429 | 3.37 | 2.09 |
| 170 | -3 | 2.3 | -0.773 | 0.433 | 3.37 | 2.1 |
| 180 | -3 | 2.3 | -0.773 | 0.436 | 3.36 | 2.1 |
| 190 | -3 | 2.3 | -0.773 | 0.439 | 3.36 | 2.1 |
| 200 | -3 | 2.3 | -0.772 | 0.442 | 3.36 | 2.1 |
| 250 | -3 | 2.31 | -0.771 | 0.452 | 3.36 | 2.1 |
| 300 | -3 | 2.31 | -0.771 | 0.457 | 3.35 | 2.1 |
| 350 | -3 | 2.31 | -0.77 | 0.46 | 3.34 | 2.1 |
| 400 | -3 | 2.31 | -0.77 | 0.462 | 3.33 | 2.09 |
| 450 | -3 | 2.31 | -0.769 | 0.462 | 3.32 | 2.09 |
| 500 | -3 | 2.31 | -0.769 | 0.462 | 3.31 | 2.08 |
| 1000 | -3 | 2.32 | -0.768 | 0.443 | 3.23 | 2.02 |
| 5000 | -3.01 | 2.33 | -0.767 | 0.299 | 2.88 | 1.74 |
| 10000 | -3.01 | 2.33 | -0.766 | 0.201 | 2.71 | 1.59 |

**Min**, minimum; **Max**, maximum; **Avg**, average.
